# Bioprospecting the microbiome of Red Sea Atlantis II brine pool for peptidases and biosynthetic genes with promising antibacterial activity

**DOI:** 10.1186/s12934-022-01835-z

**Published:** 2022-06-02

**Authors:** Laila Ziko, Omnia AbdelRaheem, Marina Nabil, Ramy K. Aziz, Rania Siam

**Affiliations:** 1 School of Life and Medical Sciences, University of Hertfordshire, Hosted by Global Academic Foundation, 11865 New Administrative Capital, Egypt; 2 Department of Biology, School of Sciences and Engineering, The American University in Cairo, 11835 New Cairo, Egypt; 3 Graduate Program of Biotechnology, School of Sciences and Engineering, The American University in Cairo, 11835 New Cairo, Egypt; 4 Department of Microbiology and Immunology, Faculty of Pharmacy, Cairo University, 11562 Cairo, Egypt; 5 Microbiology and Immunology Research Program, Children’s Cancer Hospital Egypt 57357, 11617 Cairo, Egypt; 6 University of Medicine and Health Sciences, Basseterre, West Indies Saint Kitts and Nevis

**Keywords:** Metagenomics, Natural products, Cancer, Antibiotics, Red Sea, Specialized metabolites

## Abstract

**Background:**

The search for novel antimicrobial agents is crucial as antibiotic-resistant pathogens continue to emerge, rendering the available antibiotics no longer effective. Likewise, new anti-cancer drugs are needed to combat the emergence of multi-drug resistant tumors. Marine environments are wealthy sources for natural products. Additionally, extreme marine environments are interesting niches to search for bioactive natural compounds. In the current study, a fosmid library of metagenomic DNA isolated from Atlantis II Deep Lower Convective Layer (ATII LCL), was functionally screened for antibacterial activity as well as anticancer effects.

**Results:**

Two clones exhibited antibacterial effects against the marine *Bacillus* Cc6 strain, namely clones 102-5A and 88-1G and they were further tested against eleven other challenging strains, including six safe relatives of ESKAPE pathogens (*Enterococcus faecium*, *Staphylococcus aureus*, *Klebsiella pneumoniae*, *Acinetobacter baumannii*, *Pseudomonas aeruginosa,* and *Enterobacter  *spp.), a safe relative to *Mycobacterium tuberculosis* and four resistant clinical isolates. Clone 88-1G resulted in clear zones of inhibition against eight bacterial strains, while clone 102-5A resulted in zones of inhibition against five bacterial strains. The whole cell lysates of clone 88-1G showed 15% inhibition of Mtb ClpP protease -*Mycobacterium tuberculosis* drug target-, while whole cell lysates of clone 102-5A showed 19% inhibition of Mtb ClpP protease. Whole cell lysates from the selected clones exhibited anticancer effects against MCF-7 breast cancer cells (cell viability at 50% v/v was 46.2% ± 9.9 for 88-1G clone and 38% ± 7 for 102-5A clone), U2OS osteosarcoma cells (cell viability at 50% v/v was 64.6% ± 12.3 for 88-1G clone and 28.3% ± 1.7 for 102-5A clone) and 1BR hTERT human fibroblast cells (cell viability at 50% v/v was 74.4% ± 5.6 for 88-1G clone and 57.6% ± 8.9 for 102-5A clone). Sequencing of 102-5A and 88-1G clones, and further annotation detected putative proteases and putative biosynthetic genes in clones 102-5A and 88-1G, respectively.

**Conclusions:**

The ATII LCL metagenome hosts putative peptidases and biosynthetic genes that confer antibiotic and anti-cancer effects. The tested clones exhibited promising antibacterial activities against safe relative strains to ESKAPE pathogens and *Mycobacterium tuberculosis*. Thus, searching the microbial dark matter of extreme environments is a promising approach to identify new molecules with pharmaceutical potential use.

**Supplementary Information:**

The online version contains supplementary material available at 10.1186/s12934-022-01835-z.

## Background

Antibiotic resistance is a major global health challenge, which necessitates the search for new antimicrobial agents [1]. Additionally, resistance of cancers to chemotherapy is another challenge that remains to be addressed by new anticancer agents [2]. One possible way to address such challenges is to search for natural products that have novel chemistries [3]. Particularly, searching for specialized metabolites which are produced by a subset of organisms, including microbes, is a valuable approach as it resulted into an array of compounds with antimicrobial and anticancer activities, such as doxorubicin, bleomycin, and salinosporamide [3].

Specialized metabolites are produced by a subset of organisms, and the enzymes producing them are encoded by biosynthetic gene clusters (BGCs) in the genome of the producing organism [4–6]. Currently, mining for BGCs is an expanding field, with the aim to search for antimicrobial agents to cater to the antimicrobial resistance problem, especially the ESKAPE pathogens [7, 8]. Mining for BGCs within genomes and metagenomes, followed by elucidating the chemical structure and further testing of the bioactive compound has unraveled a plethora of bioactive specialized metabolites [9, 10]. Mining for BGCs is facilitated by bioinformatics approaches, including the use of homology and machine learning algorithms [11, 12]. One approach is to exploit DNA isolated from new niches and under-explored environments, followed by functional screening for the activity of interest [13–15]. Such metagenomic approaches have enabled researchers to screen the microbial dark matter for new BGCs with antibacterial activities [16–18].

Among the marine ecological niches that harbor a huge microbial diversity are environments with extreme conditions [3, 19]. The Red Sea brine pools are areas with higher temperature and higher salinity than the rest of the Red Sea water. There are twenty-five distinct brine pools in the Red Sea [20, 21]. The largest Red Sea brine pool, possessing the highest recorded temperature and with depth of 2194 m, characterized by having large amounts of heavy metals and very low Oxygen levels, is Atlantis II Deep Red Sea brine pool (ATII) [20, 22]. The Lower Convective Layer (LCL) of the brine pool water is characteristic of multiple extreme conditions, as it has a salinity of 270 psu, a pH of 5.3, in addition to the high temperature of 68.2°C [20, 22]. In this work, we used a previously generated fosmid library from the microbial metagenome of ATII LCL [23, 24] and tested two selected clones for antibacterial and anticancer activity, in addition to sequencing and annotating the genes inserted in these two clones.

Earlier, we have screened the metagenomic fosmid library that harbored prokaryotic environmental DNA from the Atlantis II Red Sea brine pool LCL water samples (ATII) [23, 24]. In our earlier study, we focused on two clones that harbored putative orphan biosynthetic gene clusters [24]. We focused this current study on two other clones, namely 88-1G and 102-5A, for antibacterial effects against clinically relevant strains and assessed their cytotoxic activities against different mammalian cell lines.

The aim of this work was to screen the ATII LCL metagenomic fosmid library by a functional assay for anti-bacterial effects. Two positive clones were then selected to assess their antibacterial effects against clinically relevant bacterial strains. Furthermore, cytotoxic effects of the extracts from the two clones were determined against breast cancer and bone cancer cells in addition to non-cancerous fibroblasts. Lastly, the selected clone inserts were sequenced, and their putative protein-coding genes were annotated. The present two clones harbored individual genes which resulted in the observed effects.

## Results

### Antibacterial effects of clones 88-1G and 102-5A from Red Sea Atlantis II LCL (or Red Sea ATII LCL) fosmid library against clinically relevant strains

In antimicrobial overlay assays, clones 88-1G and 102-5A exhibited zones of inhibition against the challenging strain *Bacillus* Cc6. The antibacterial overlay assay was also conducted against eleven additional challenging strains (Table 1), including *Staphylococcus epidermidis*, *Erwinia carotovora, Enterococcus raffinosus*, *Acinetobacter baylyi, Enterobacter aerogenes, Mycobacterium smegmatis, Pseudomonas putida,* and the following clinical isolates: MRSA-ZC1, MRSA-ZC2, MRSA-ZC6, and *Acinetobacter* ZC2. Clone 88-1G showed zones of inhibition against nine strains: *Staphylococcus epidermidis*, *Erwinia carotovora, Enterococcus raffinosus, Enterobacter aerogenes, Mycobacterium smegmatis, Acinetobacter* ZC2, MRSA-ZC1, MRSA-ZC2*,* and *Bacillus* Cc6 (Additional file 1: Fig. S1). Clone 102-5A showed zones of inhibition against six of the tested strains, namely *Staphylococcus epidermidis*, *Mycobacterium smegmatis*,  *Pseudomonas putida, Acinetobacter* ZC2*,* MRSA-ZC6*,* and *Bacillus* Cc6 (Additional file 1: Fig. S2).

**Table 1:** The antibacterial effect of clones 88-1G and 102-5A against the challenging strains

	Name of the Strain		88-1G	102-5A
Marine strain				
A	*Bacillus* Cc6		+	+
Safe relatives of ESKAPE pathogens				
	Safe strain	Relative Pathogen		
B	*Staphylococcus epidermidis*	*Staphylococcus aureus*	+	+
C	*Erwinia carotovora*	*Erwinia species*	+	–
D	*Enterococcus raffinosus*	*Enterococcus faecium*	+	–
E	*Enterobacter aerogenes*	*Enterobacter species*	+	–
F	*Mycobacterium smegmatis*	*Mycobacterium tuberculosis*	+	+
G	*Pseudomonas putida*	*Pseudomonas aeruginosa*	–	+
H	*Acinetobacter baylyi*	*Acinetobacter baumanni*	–	–
Clinical isolates				
I	Resistant ZC- AB2		+	+
J	MRSA-ZC1		+	–
K	MRSA-ZC2		+	–
L	MRSA-ZC6		–	+

*The detection of an inhibition zone is denoted by* + *and absence of the inhibition zone is denoted by –*

### Whole cell lysates from 88-1G and 102-5A Red Sea Atlantis II LCL (or Red Sea ATII LCL) fosmid library clones showed inhibition of *M. tuberculosis* target ClpP Protease

**Fig. 1.**
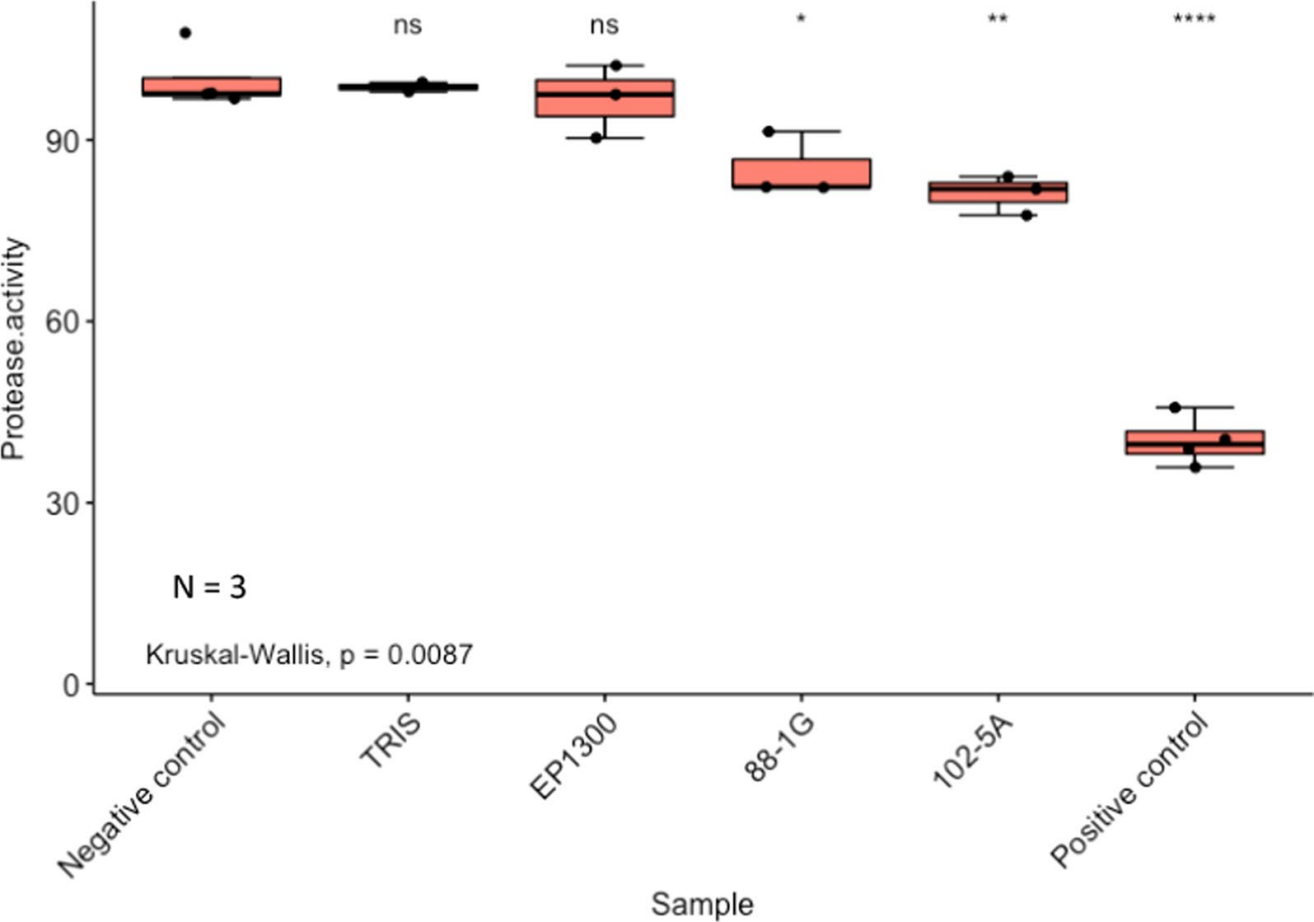
88-1G and 102-5A clone extracts inhibit the peptidase activity of Mtb ClpP1P2. ClpP protease % activity was analyzed by Mtb ClpP assay, and fluorescence was obtained. The statistical significance was estimated by the Kruskal–Wallis test followed by post hoc Wilcoxon tests. p-values are denoted as follows: *p-value ≤ 0.05, **p-value < 0.01, and ****p-value ≤ 0.0001

Using the ClpP protease assay to assess the effectiveness of the clone extracts against *Mycobacterium tuberculosis*, the 88-1G extract showed ~ 15% inhibition of Mtb ClpP1P2 peptidase activity. This inhibition was statistically significant when compared to the negative controls: TRIS (the extraction solvent) and EPI300 control. The 102-5A fosmid extract was slightly more potent and demonstrated an inhibition activity equivalent to 19% that was also statistically significant when compared to the negative controls (Fig. 1).

### Differential cytotoxicity of the extracts from 88-1G and 102-5A Red Sea Atlantis II LCL (or Red Sea ATII LCL) fosmid library clones against cancer cell lines

**Fig. 2:**
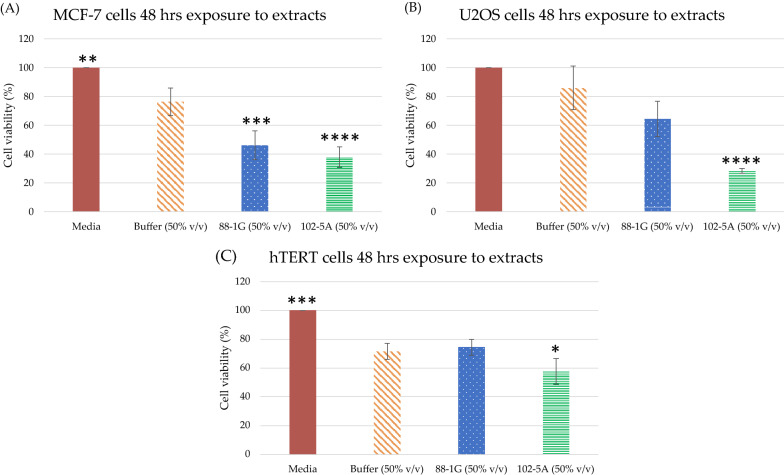
Cell viability assay results. The cell viability percentage after incubation with the fosmid cell lysates: (**A**) MCF-7 cells, (**B**) U2OS cells, and (**C**) 1BR hTERT cells viability, after 48 h exposure to whole-cell extracts from clones 88-1G (blue) and 102-5A (Green). A 50% v/v concentration of the prepared whole-cell extracts was used. The results were obtained from at least three independent experiments and the p values were calculated in comparison to negative control cells exposed to the buffer concentration 50% v/v. p-values are denoted as follows: * ≤ 0.05, ** ≤ 0.01, ***≤ 0.001 and ****≤ 0.0001

All the extracts were prepared similarly, and the cytotoxic effects were determined for equal concentrations of whole-cell lysates against MCF-7, U2OS cancer cell lines, and 1BR hTERT cells for 48 h. (Fig. 2). Extracts of both 88-1G and 102-5A clones exhibited significant cancer cell cytotoxic effects at 50% v/v concentration. Whole-cell lysate extracts at 50% v/v of clone 102-5A were more cytotoxic to all the cell lines when compared to 88-1G extracts (Fig. 2). For the breast cancer cell line MCF-7 cells, the cell viability was 46.2% ± 9.9 and 38% ± 7 after 48 h exposure to 88-1G and 102-5A clone whole cell lysate extracts, respectively (Fig. 2A). For the osteosarcoma cell line U2OS cells, the cell viability was 64.6% ± 12.3 and 28.3% ± 1.7 after 48 h exposure to 88-1G clone and 102-5A whole cell lysate extracts, respectively (Fig. 2B). Lastly, for the human skin fibroblasts 1BR hTERT cells, the cell viability was 74.4% ± 5.6 and 57.6% ± 8.9 after 48 h exposure to 88-1G and 102-5A whole cell lysate extracts, respectively (Fig. 2C).

### Annotated sequences from 88-1G and 102-5A Red Sea Atlantis II LCL (or Red Sea ATII LCL) fosmid library clones are similar to genes from organisms inhabiting similar extreme niches

The fosmid DNA was extracted from both clones (102-5A and 88-1G) and sequenced in an Illumina MiSeq platform. After trimming the vector and *E. coli* sequences, 102-5A had 8 scaffolds and 88-1G had 19 scaffolds. The assembly metrics are denoted in Table 2.

**Table 2:** Assembly metrics of fosmid DNA sequencing for inserts in clones 88-1G and 102-5A

Fosmid	Sequencing method	De novo assembly program	Total bases	Total reads	Total reads after quality filtering	Total bases in scaffolds	Scaffold N50	Scaffolds No	After filtering & trimming				Number of scaffolds with RAST annotated PEGs
									Scaffolds No	Bases No	N50	PEGs No	
88-1G	Illumina MiSeq: 300 bp paired—end read (Illumina MiSeq V3)	De novo assembly with CLC Genomics Workbench v 8.0	72,271,200	240,904	240,836	506,285	1,284	369	19	16,457	2,564	22	6
102-5A	Illumina MiSeq: 300 bp paired—end read (Illumina MiSeq V3)	De novo assembly with CLC Genomics Workbench v 8.0	73,839,600	246,132	246,080	1,213,995	1,478	800	8	17,793	12,037	21	2

After the vector and host sequences were trimmed, the number of PEGs (protein-encoding genes) detected by RAST (Rapid Annotations using Subsystems Technology) in each assembly was 21 and 22 in inserts 102-5A and 88-1G, respectively (Table 2). The majority of PEGs detected by RAST coded for hypothetical proteins. This includes 81% and 86% in 102-5A PEGs and 88-1G, respectively (Additional file 1: Tables S1, 2). To further annotate the PEGs, we analyzed each PEG by PSI-BLAST (Position-Specific Iterative Basic Local Alignment Search Tool) to determine the most similar sequences in publicly available databases. The full annotation results for all the detected PEGs are presented in Additional file 1: Tables S1 and 2. PEGs with hits in PSI-BLAST of higher than the threshold E-value 0.005 were depicted as non-significant (Table 3, Additional file 1: Tables S1, Tables S2).

**Table 3:** Selected PEGs on 88-1G and 102-5A clone inserts

	Predicted function	Scaffold	Start	Stop	PEG	RAST annotation	Best hit from PSI-BLAST	Query coverage	E-value	Identity%	Accession number
88-1G	Transcription regulators	Scaffold_11	51	194	1	Hypothetical protein	LysR family transcriptional regulator [*Hydrocarboniphaga effusa*]	93%	4.9	36%	gi|494335651|WP_007184865.1
		Scaffold_13	2702	2971	10	Hypothetical protein	CopG family transcriptional regulator [*Natronococcus occultus*]	80%	0.001	35%	gi|505136118|WP_015323220.1
	Biosynthetic gene	Scaffold_8	1248	466	18	Glycosyltransferase	Hypothetical protein AUJ13_03040 [*Candidatus Micrarchaeota* archaeon CG1_02_49_24]	81%	6e−21	30%	gi|1101062238|OIO23950.1
	Peptidase	Scaffold_8	1548	2609	20	Peptidase S8 and S53	Serine protease AprX [*Bradyrhizobium erythrophlei*]	79%	6e−26	34%	gi|1119071599|SIO25423.1
102-5A	Peptidases	Scaffold_1	4542	4733	6	Hypothetical protein	S9 family peptidase [*Nocardia pseudobrasiliensis*]	68%	0.79	49%	gi|1056574138|WP_068006013.1
		Scaffold_1	6517	7809	11	Subtilisin precursor	Peptidase S8 [*Streptomyces* sp. CB02613]	75%	3e-21	28%	gi|1288530123|WP_100563527.1
	Hydrolase	Scaffold_1	8840	8697	13	Hypothetical protein	Cobalt-precorrin 5A hydrolase [*Hyphomicrobium facile*]	93%	0.56	46%	gi|1097765764|SFV26573.1

Annotation results of clones 102-5A and 88-1G showed similarity to organisms with significant hits, which are inhabiting similar environments as the Red Sea Atlantis II brine pool. Protein-based phylogeny for clone 102-5A resulted in similarity to organisms such as *Candidatus Bathyarchaeota archaeon* ex4484_40, an uncultured hydrothermal sediment archaeon from the Guaymas basin in California [25], candidate division *MSBL1 archaeon* SCGC-AAA382A20, uncultured archaea from the Red Sea Neurus brine [26], *Thermoanaerobacterium *sp. PSU-2, a thermophilic bacterium [27], and *Candidatus Nanosalinarum *sp. J07AB56, a halophilic archaeon [28]. MIBiG (Minimum Information about a Biosynthetic Gene cluster) alignments yielded one significant result, which was evident by the alignment of a putative S8 peptidase with the SLI-2138 RiPP (ribosomally synthesized and post-translationally modified peptide) biosynthetic gene cluster [29, 30]. Similarly, clone 88-1G protein-based phylogeny detected significant similarity with: *Candidatus Micrarchaeota archaeon* CG1_02_49_24, an uncultured archaeon adapted to high carbon dioxide levels [31], and the haloalkaliphilic archaea *Natronococcus occultus* [32] and *Natronobacterium texcoconense * [33]. MIBiG comparison yielded two significant results, which was evident by the alignment of a putative serine protease AprX with the SLI-2138 biosynthetic gene cluster, and the alignment of a putative glycosyltransferase with a glucosyltransferase within a carotenoid biosynthetic gene cluster [29].

### Annotation of putative biosynthetic genes inserted in clones 102-5A and 88-1G

**Fig. 3:**
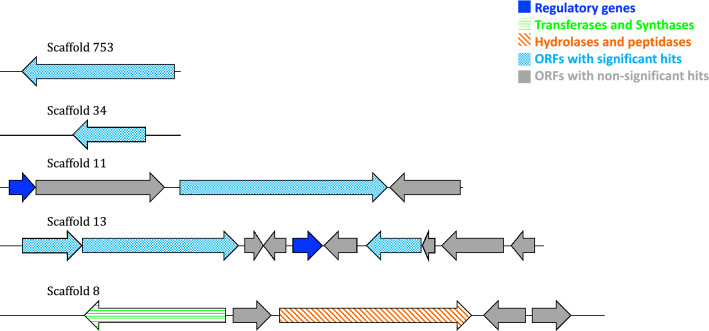
Sequence maps of the putative biosynthetic genes on 88-1G clone insert. Five of the six annotated scaffolds are depicted. Top to bottom: scaffold 753, scaffold 34, scaffold 11, scaffold 13 and scaffold 8. Dark blue: regulatory genes. Green: Transferases and synthases. Orange: hydrolases and peptidases. Blue: ORFs with significant hits. Grey: ORFs with non-significant hits

Clone 88-1G insert harbored the following PEGs (Fig. 3, Table 3): (1) a putative LysR family transcriptional regulator [*Hydrocarboniphaga effusa*], (2) a putative CopG family transcriptional regulator [*Natronococcus occultus*], (3) a putative serine protease AprX [*Bradyrhizobium erythrophlei]* and (4) a putative glycosyltransferase [*Candidatus Micrarchaeota archaeon* CG1_02_49_24].

**Fig. 4:**
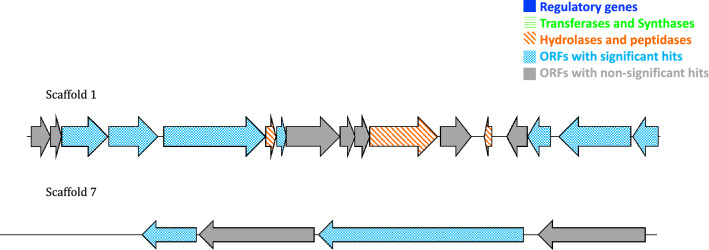
Sequence maps of the putative biosynthetic genes on 102-5A clone insert. All two annotated scaffolds are depicted. Top to bottom: scaffold 1 and scaffold 7. Dark blue: regulatory genes. Green: Transferases and synthases. Orange: hydrolases and peptidases. Blue: ORFs with significant hits. Grey: ORFs with non-significant hits

Annotation of clone 102-5A insert revealed two putative peptidases and a putative hydrolase, with the best hits being (Fig. 4, Table 3): (1) S9 family peptidase [*Nocardia pseudobrasiliensis*], (2) peptidase S8 [*Streptomyces* sp. CB02613] and (3) cobalt-precorrin 5A hydrolase [*Hyphomicrobium facile*].

## Discussion

### Broad spectrum antibacterial activity of clones from Red Sea brine pool Atlantis II (ATII) LCL fosmid library

Clone 88-1G exhibited antibacterial activity against *Bacillus* Cc6 marine strain, which is a Gram-positive marine bacterial strain (Table 1, Additional file 1: Fig. S1), implying its possible in situ activity, as the metagenomic clones were from a marine environment [34]. Additionally, antibacterial activity was also observed against clinically relevant strains including *Staphylococcus epidermidis*, *Erwinia carotovora, Enterococcus raffinosus* and *Enterobacter aerogenes*, which are safe relatives of ESKAPE pathogens (Table 1, Additional file 1: Fig. S1). Methicillin-resistant *Staphylococcus aureus* (MRSA) and *Acinetobacter* (*Acinetobacter* AB2) clinical isolates were obtained from hospitalized patients in Egypt. Clone 88-1G inhibited the growth of *Acinetobacter* AB2 strain, MRSA-ZC1, and MRSA-ZC2. Additionally, clone 88-1G resulted in an inhibition zone with the acid-fast bacteria *Mycobacterium smegmatis*. This result was corroborated with the results of the Mtb ClpP protease assay [30, 31], as the whole-cell lysate of 88-1G resulted in 15% inhibition of the *M. tuberculosis* target enzyme.

Clone 102-5A similarly exhibited antibacterial activity against *Bacillus* Cc6 marine strain (Table 1, Additional file 1: Fig. S2) [24, 34], however it showed a broad but different pattern of zones of inhibition than the one observed for 88-1G clone. Antibacterial activity was also observed against *Staphylococcus epidermidis* and *Pseudomonas putida*, other safe relatives of ESKAPE pathogens (Table 1, Additional file 1: Fig. S2). Antimicrobial activities against the ESKAPE pathogens are important because of their emerging antimicrobial resistance, particularly in nosocomial settings [32, 33]. Clone 102-5A inhibited the growth of *Acinetobacter* AB2 strain and MRSA-ZC6. Additionally, it displayed an inhibition zone against *Mycobacterium smegmatis* (Table 1, Additional file 1: Fig. S2). We confirmed the former results by performing an Mtb ClpP protease assay [35, 36]. The whole-cell lysates of 102-5A resulted in 19% inhibition of the *M. tuberculosis* target enzyme, which is higher inhibition than that observed for lysates of 88-1G clone.

In conclusion, each of the tested clones exhibited a unique and broad antibacterial activity pattern, including gram-positive, gram-negative strains, and acid-fast bacteria.

### Anticancer activity of whole-cell lysates from the Red Sea brine pool Atlantis II (ATII) LCL fosmid library clones

We observed differential cytotoxicity of the two fosmid library clones against the different tested cell lines. For the breast cancer cell line MCF-7, the whole cell lysate extracts of clone 102-5A exhibited a greater cytotoxic effect with cell viability (38% ± 7), when compared to the 88-1G clone, which exhibited a cell viability of 46.2% ± 9.9 (Fig. 2A). 102-5A extracts also exhibited more cytotoxicity against U2OS cells -osteosarcoma cell line- (28.3% ± 1.7) when compared to extracts from the 88-1G (64.6% ± 12.3) (Fig. 2B).

The cell viability of 1BR hTERT cells incubated with clone 88-1G extracts was close to that of the negative control: 74.4% ± 5.6 cell viability and 71.6% ± 5.6 cell viability with 88-1G extracts and buffer control, respectively. On the other hand, 102-5A cell lysates were more lethal to the 1BR hTERT cells (57.6% ± 8.9 viability) (Fig. 2C). The lack of cytotoxicity observed on incubation of 1BR hTERT with 88-1G suggests that 88-1G is a better candidate for selective cancer cytotoxicity.

### Putative peptidases and hydrolase detected on clone 102-5A insert of the Red Sea brine pool Atlantis II (ATII) LCL

Clone 102-5A annotation included putative (Fig. 4, Table 3): (1) S9 peptidase, (2) S8 peptidase and (3) a hydrolase. This includes a serine protease of the S9 peptidase family, whose members are not yet fully characterized [37]. A novel putative hydrolase was identified by a similar approach from the metagenomes of microbes associated with marine sponges and was observed to exhibit antibacterial effects [34]. It was hypothesized that hydrolases were produced by eukaryotic hosts to limit the overgrowth of the microbial symbionts [34]. Although the microbial community included in the present study is not a symbiotic community owing to the studied sample, however, it is likely that the detected putative hydrolytic enzymes would have similar antagonistic effects owing to the observed antibacterial activities.

Putative proteases with antibacterial effects were detected in a study using *Ralstonia* species as the host for heterologous gene expression, rather than *E. coli *[15]*.* The detected putative proteases belonged to the metallopeptidase family and the S8A serine protease family [15]. Such antibacterial properties of proteases are likely to act on breaking the amide bond connecting two adjacent present peptidoglycan layers within the cell walls of both gram-positive and gram-negative bacteria, which could explain the broad spectrum of activity observed in this study [15]. Previous studies have reported the antibacterial and anticancer activities of peptidases [15, 38, 39]. It is worth noting that S8 peptidases (subtilases) have industrial significance including the high-alkaline subtilases (family A), which are used in the detergent industry [40].

The annotated peptidases and hydrolase may play a role in the observed antibacterial and cytotoxic effects, particularly that two putative serine peptidases were detected on 102-5A clone insert; namely an S9 and an S8 peptidase, in addition to a putative hydrolase. Further structure and function analysis studies need to be performed to confirm the hypothesized roles of the annotated peptidases and hydrolase in the antibacterial and cytotoxic effects and to pinpoint the inhibitory role of each protein.

### Putative biosynthetic gene and peptidase detected on clone 88-1G insert of the Red Sea brine pool Atlantis II  (ATII) LCL

Clone 88-1G fosmid DNA harbored several putative biosynthetic genes linked to the observed activities (Fig. 3, Table 3). Four genes were detected that are likely to contribute to the observed activities: (1) a serine protease, (2) a glycosyltransferase and (3) two putative transcriptional regulators. A serine protease was detected, and as aforementioned, it is likely to function by targeting the synthesis of the peptidoglycan bacterial cell wall [15].

Furthermore, glycosyltransferases have been reported to play a pivotal role in specialized metabolite biosynthetic pathways [11, 41]. Glycosyltransferase enzymes catalyze the formation of glycosidic bonds and the transfer of sugar moieties to carbohydrate, lipid, small molecule, protein or DNA substrates [42]. Previous studies had identified the role of glycosyltransferases in the synthesis of natural products [43]. Further biochemical studies need to be conducted to elucidate the natural product produced by the putative glycosyltransferase enzyme and its role in the observed antibacterial and/or cytotoxic effects.

### Future prospects

Our present study points to the importance of bioprospecting microbial dark matter from extreme environments, particularly in the discovery of antibacterial and anti-cancer agents. Yet the present study use of a mesophilic bacterial strain in the fosmid library construction is likely to limit the expression of extremophilic genes [8] and therefore further studies should assess the activity in extremophiles.

Additionally, future studies should aim to identify the nature of the antibacterial and/or anticancer active compounds, whether it is a specialized metabolite, or a protein as per the annotations. Peptide active compounds can be challenging in drug delivery [44]. However, several studies are considering AMPs (antimicrobial peptides), RiPPs, bacteriocins and other peptides for anti-bacterial and anti-cancer testing, to increase the array of pharmaceutically-relevant natural products as drug leads [9, 44]. Further structure–function studies would also be interesting in elucidating the cytotoxic and antibacterial cellular mechanisms of the active compound/s in the clones.

## Conclusions

Mining microbial metagenomes for specialized metabolites with pharmaceutical use is one way to find new antibacterial and anticancer compounds. Through the approach of mining yet under-explored environments of microbiomes inhabiting harsh marine niches, followed by functional screening, putative peptidases and biosynthetic genes have been identified and further studies are required to fully decipher the specialized metabolites’ structures.

## Methods

### Metagenomic fosmid library screening for antibacterial activity

Water samples from the lower Convective Layer (LCL) of Atlantis II Red Sea brine pool (21° 20.72’ N and 38° 04.59’ E) was previously collected in the 2010 KAUST/WHOI/HCMR expedition [23]. Prokaryotic eDNA was extracted from the 0.1 µm filter as previously described [45]. The ATII LCL fosmid library was previously constructed as per the manufacturer’s protocol and it was composed of 10,656 clones, by using pCC2FOS vector with the CopyControl Fosmid Library Production Kit (Epicenter) [23]. A fresh copy of the aforementioned fosmid library was prepared prior to the downstream assays and further used [24]. The recipient strain for the fosmid was EPI300-T1^R^ *E. coli* as per the manufacturer’s protocol.

### Antimicrobial overlay assay

An antimicrobial overlay assay was used to test for clones with anti-bacterial activity similar to that reported in the literature [34, 46]. *E. coli* CBAA11 strain producing the antibacterial tambjamine was used as the positive control strain [13, 34]. The challenging strain that was used was *Bacillus* Cc6 strain which is a marine *Bacillus* strain associated with the Australian marine sponge *Cymbastela concentrica* (gift from Prof. Torsten Thomas, University of New South Wales) [13, 34]. *Bacillus* Cc6 strain was used as a challenging strain because it was isolated from a marine environment and because it was reported in a functional screening assay utilizing the same vector CC2 as used in our study [13, 34]. *E. coli* CBAA11 strain was chosen as a positive control because it produces the antibacterial tambjamine and was reported to result in clear zones against *Bacillus Cc6* [13, 34]. All the plates were supplemented with 0.01% arabinose and 12.5 µg/ml chloramphenicol. All the plates were tested against the clones and included both a positive control (*E.coli* CBAA11) and a negative control (EPI300).

First the clones were plated on LB/chloramphenicol/arabinose overnight at 37 °C. Chloramphenicol was not added to the other strains, other than *Bacillus* Cc6 strain. The following day, the plates were incubated overnight at 25 °C and an overnight culture of *Bacillus CC6* in LB/chloramphenicol was prepared and incubated at 37 °C with shaking. The following day, the starter culture was used to inoculate 100 ml LB/chloramphenicol of *Bacillus CC6* till the OD reached 0.5. Then *Bacillus CC6* was poured on the plates using top agar at 1:1000 dilution. The overlaid plates were finally incubated overnight at 25 °C and observed carefully for clear zones in the top layer [34]. Clones exhibiting antibacterial activity were further tested against the eleven challenging strains (Table 1).

### Extract preparation

Whole-cell extracts were prepared from the clones namely 102-5A and 88-1G, using a method similar to that reported in the literature [47]. Overnight cultures from the *E. coli* bearing the eDNA from the positive clones -previously supplemented with auto-induction solution and chloramphenicol- were centrifuged at 3,500 rpm for 10 min. Afterwards, the cell pellets were resuspended in 0.01 M Tris–HCl pH 7. The extracts were then sonicated on ice at 20% maximal amplitude for 370 s (with 10 s intervals without sonication). Finally, the extracts were sterile-filtered with 0.2 µm membrane filters (Corning) [47]. Two negative controls were used: (1) cells incubated with only the media, and (2) cells incubated with buffer. The buffer concentration, in the latter, was the same concentration used in the highest extract concentration 50% v/v (Fig. 2).

### Cell lines and culture conditions

Three cell lines were used for the cytotoxicity assay, a human breast adenocarcinoma cell line (MCF-7) [48], an osteosarcoma cell line (U2OS) [49] (gift from Dr. Andreas Kakarougkas (University of Sussex) and skin fibroblast cells–wild-type and non-cancerous cells- that are immortalized with human telomerase reverse transcriptase (1BR hTERT) [50–52]. The cells were cultured in DMEM (Lonza, Germany), supplemented with 10% Fetal Bovine Serum (Lonza, Germany) and 5% Penicillin–Streptomycin (Lonza, Germany). All cells were grown in an incubator adjusted at 37°C and 5% CO_2_ at a seeding density of 10^4^ cells/well.

### Cell viability assay

The initial seeding density was adjusted to 10^4^ cells/well and left overnight to adhere to the bottom of the 96-well plates (Greiner Bio-One, Germany). The old media was discarded the following day and the media containing the different v/v% concentrations of the extracts was added (0, 15, 20 and 50%). The cells were exposed for 48 h to the extracts before assessing the cell viability.

The extracts were incubated for 48 h with MCF-7, U2OS and 1BR hTERT cell lines with different v/v% concentrations (0, 15, 20 and 50%) [47]. Cell viability percentage was assessed by the MTT assay after 48 h exposure to the extracts. Firstly, the media was replaced by 100 µl fresh media containing MTT reagent 3-(4, 5-dimethylthiazolyl-2)-2, 5-diphenyltetrazolium bromide in a concentration of 20 µl MTT (5 mg/ml) (Serva, Germany) for 3 h. Afterwards, the media was discarded and 100 µl DMSO (Sigma-Aldrich, USA) was added to solubilize the purple precipitates.

Several negative controls were used in the experiments. Cells with no cell lysates added were used as the first negative control cells. Cells exposed to the buffer only with no cell lysates were used to test the buffer effect. Additionally, cells exposed to the cell lysates of EPI300 strain were used to test the effect of the strain.

All the negative control cells (A_595_ control) were supplemented with complete media. The wells used for measuring A_595_ blank were only supplemented with media and no cells. Absorbance was finally measured at 595 nm (A_595_), using a SPECTROstar Nano microplate reader (BMG LabTech, Germany). Cell viability was calculated as follows:

$${\text{Cell Viability }}\% = \, \left[ {\left( {{\text{A}}_{{{595}}} {\text{sample }}{-}{\text{ A}}_{{{595}}} {\text{blank}}} \right)/\left( {{\text{A}}_{{{595}}} {\text{control }}{-}{\text{ A}}_{{{595}}} {\text{blank}}} \right)} \right] \times { 1}00$$

### Sequencing and bioinformatics

The two clones, 88-1G and 102-5A, were selected for DNA extraction and sequencing [24]. Overnight cultures were supplemented with auto-inducer/chloramphenicol. Fosmid DNA was extracted by QIAprep Spin Miniprep Kit (Qiagen). The fosmid DNA was sequenced using Illumina MiSeq V3 300 bp paired-end read platform (LGC, Germany). After sequencing and quality filtering, the reads were assembled by de novo assembly programs, namely SOAPdenovo2 [53] and CLC Genomics Workbench v 8.0 (Qiagen), respectively. The details of the sequencing and assembly metrics are depicted in Table 2.

Prior to annotation, the vector sequences—pCC2FOS™- were trimmed from the resulting scaffolds. *E. coli* sequences were also removed from the sequences. NC_010473 DH10B reference sequence was used because the strain used for the fosmid library construction—EPI300™-T1R Phage T1-resistant *E. coli*- was derived from *E. coli* DH10B strain. The resulting scaffolds were used to determine the putative PEGs –protein-encoding genes- using RAST platform (analysis conducted March 2017) [54]. Each PEG was further compared to sequences in the publicly available databases by using PSI-BLAST [55]. The PEGs were also compared to the protein sequences curated in the Minimum Information about a Biosynthetic Gene cluster (MIBiG) database by BLASTX [30]. Lastly, PFAM alignments were done for the selected PEGs (Additional file 1: Fig. S4).

### *M. tuberculosis* ClpP Protease Assay

The protein degradation enzyme ClpP protease of *M. tuberculosis* that is composed of subunits P1 and P2 is a proven drug target against *M*. *tuberculosis*. Mtb ClpP assay was used to determine the inhibition of the peptidase activity of Mtb ClpP1P2. The cleavage of the labeled peptide substrate generates fluorescence at 460 nm (excitation at 355 nm). The assay was performed in a F-bottom, black plate, clear bottom 96- well plate with a final reaction volume of 30 µl, as per the manufacturer's instructions (ProFoldin, USA). The assay buffer and Mtb ClP1P2 were mixed. For the negative control, DMSO was used, and for the positive control, the Mtb ClP1P2 was boiled at 95 °C for 10 min (35, 36). Finally, for the extracts under investigation, 0.6 µl of 450 µg/mL of 88-1G, and 102-5A were added and incubated at 37 °C for 20 min. The 100 X labeled substrate was freshly diluted to make the 10 X, added to the assay reaction solution, and set again at 37°C for 60 min. The fluorescence was measured using FLUOstar OPTIMA microplate reader (BMG LabTech, Germany) by exciting at 355 nm and following the increase in fluorescence emission at 460 nm. Percentage inhibition was calculated = F (sample)/F (control) × 100.

## Statistical analysis

Statistical analysis was conducted by one-way ANOVA and Post-hoc Tukey test to calculate the *P* values for the cytotoxicity results (Fig. 2). R program version 4.1.2 (R Development Core Team 2016) was used to compute the statistical calculations. The *P* values represent the significant differences between the mean of each condition and the mean of the negative control cells with buffer concentration 50% v/v (* ≤ 0.05, ** ≤ 0.01, *** ≤ 0.001 and **** ≤ 0.0001).

R program was also used to generate Fig. 1, and Kruskal–Wallis one-way analysis of variance (ANOVA) was used to compute the p-values.

## Supplementary Information

**Additional file 1: Figure S1**. The anti-bacterial overlay assay results of 88-1G fosmid clone, **Figure S2.** The anti-bacterial overlay assay results of 102-5A fosmid clone, **Figure S3.** Negative control for the overlay assay. **Figure S4.** The alignment of the PFAM hits of the selected PEGs in 88_1G and 102_5A clones. **Table S1.** Annotation of all PEGs after filtering and trimming of the putative orphan biosynthetic gene clusters on 88-1G clone insert, **Table S2.** Annotation of all PEGs after filtering and trimming of the putative orphan biosynthetic gene clusters on 102-5A clone insert.

## Data Availability

The datasets of generated and analysed during the current study, comprising all scaffold sequences and their corresponding RAST annotations, are available with GenBank Accession Numbers BankIt2431428 OK545880-OK545898 for 88-1G, and BankIt2509949 OK545899-OK545906 for 102-5A.
