# Supplementary material for: Bioprospecting the microbiome of Red Sea Atlantis II brine pool for peptidases and biosynthetic genes with promising antibacterial activity
Source: Microb Cell Fact. 2022 Jun 2;21:109. doi: 10.1186/s12934-022-01835-z (PMC9161539; doi:10.1186/s12934-022-01835-z)
Supplement: Supplementary file 1 — Additional file 1: Figure S1. The anti-bacterial overlay assay results of 88-1G fosmid clone, Figure S2. The anti-bacterial overlay assay results of 102-5A fosmid clone, Figure S3. Negative control for the overlay assay. Figure S4. The alignment of the PFAM hits of the selected PEGs in 88_1G and 102_5A clones. Table S1. Annotation of all PEGs after filtering and trimming of the putative orphan biosynthetic gene clusters on 88-1G clone insert, Table S2. Annotation of all PEGs after filtering and trimming of the putative orphan biosynthetic gene clusters on 102-5A clone insert. [file 12934_2022_1835_MOESM1_ESM.pdf]

Additional file

Figure S1. Anti-bacterial overlay assay of 88-1G fosmid clones.

Results of the overlay are depicted. The bacterial strains exhibiting zones of inhibitions are presented and include (A) *Bacillus* Cc6 (B) *Staphylococcus epidermidis* (C) *Erwinia carotovora* (D) *Enterobacter raffinosus* (E) *Enterobacter aerogenes* (F) *Mycobacterium smegmatis* (I) Resistant ZC- AB2 (J) MRSA-ZC1 (K) MRSA-ZC2.

A 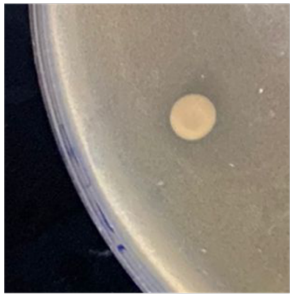	B 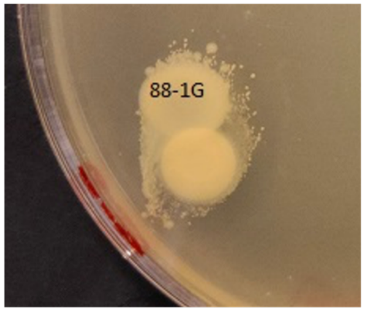	C 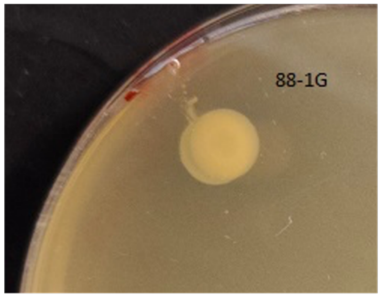
Bacillus Cc6	*Staphylococcus epidermidis*	*Erwinia carotovora*
D 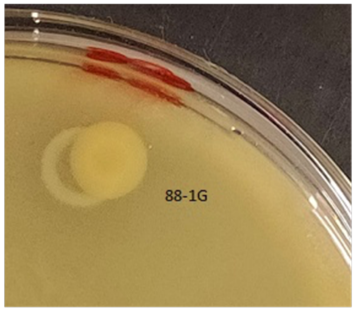	*E* 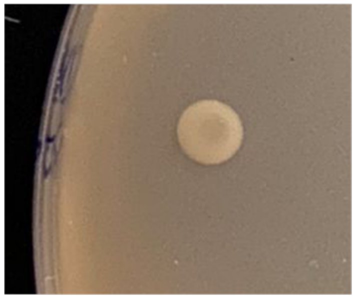	*F* 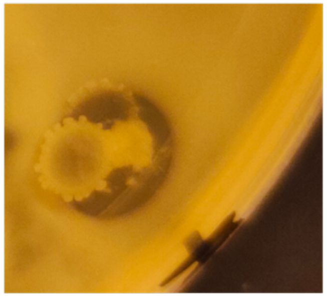
Enterococcus raffinosus	*Enterobacter aerogenes*	*Mycobacterium smegmatis*
I 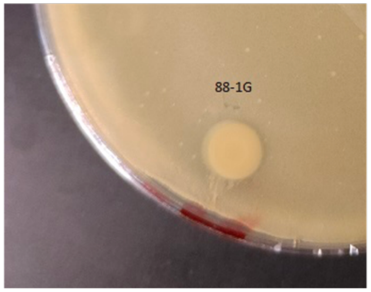	*J* 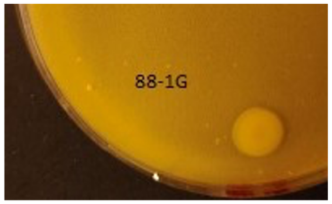	*K* 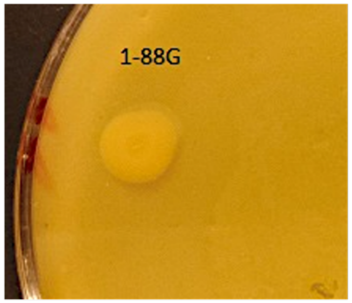
Resistant ZC- AB2	*MRSA-ZC1*	*MRSA-ZC2*

Figure S2. Anti-bacterial overlay assay of 102-5AG fosmid clones.

Results of the overlay are depicted. The strains exhibiting zones of inhibitions are shown and includes (A) *Bacillus* Cc6 (B) *Staphylococcus epidermidis* (F) *Mycobacterium smegmatis* (G) *Pseudomonas putida* (I) Resistant ZC- AB2 (L) MRSA-ZC6.

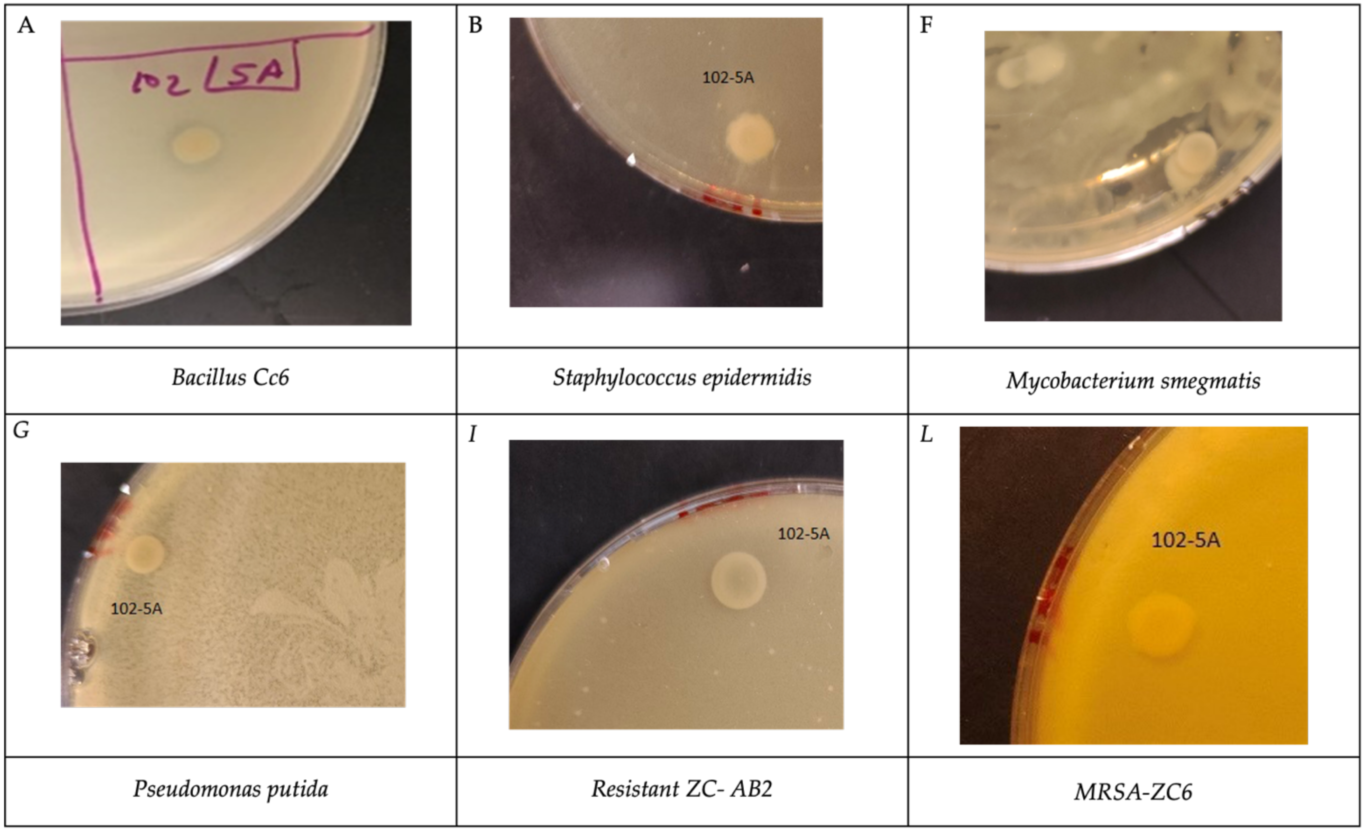

Figure S3 Negative control for the overlay assay.

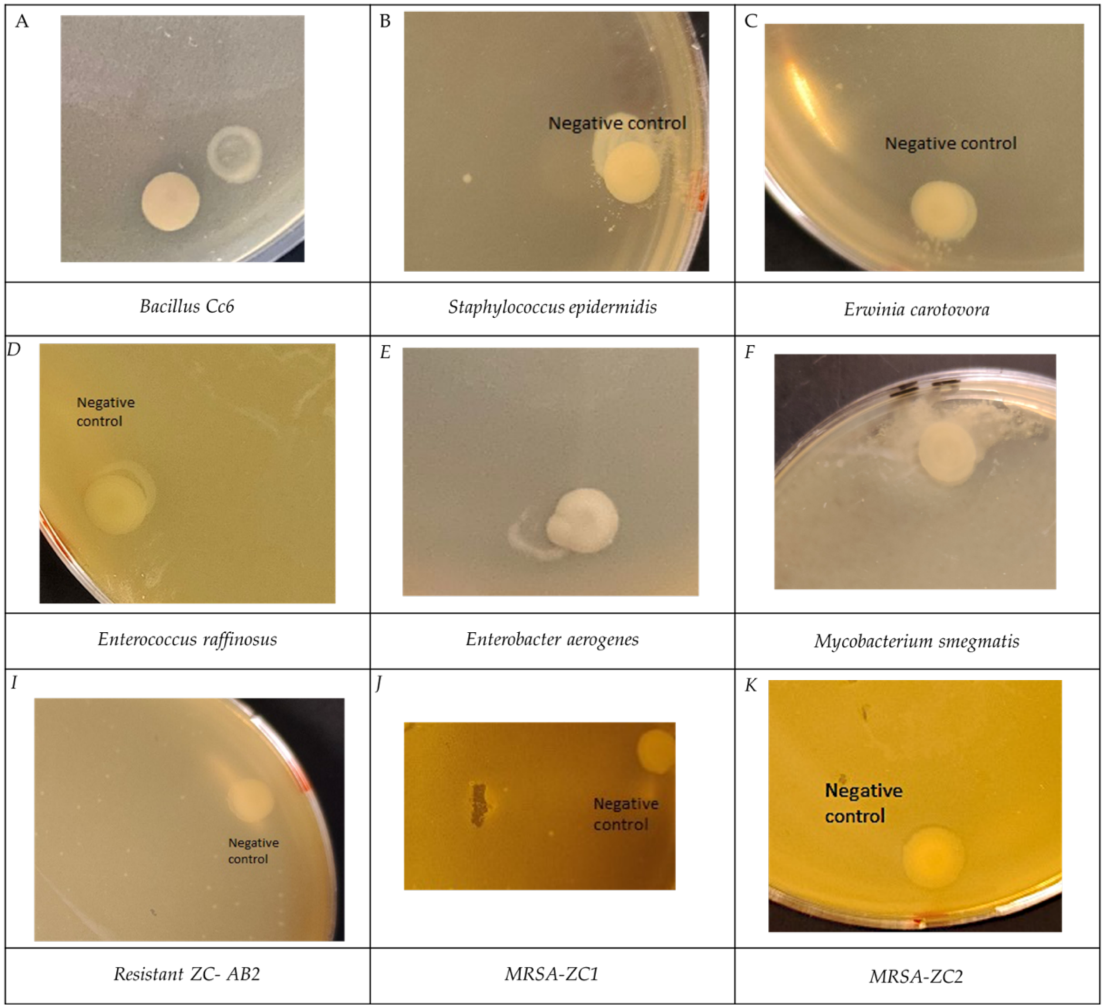

Figure S4. The alignment of the PFAM hits of the selected PEGs in 88_1G and 102_5A clones.

Hits and alignments of PFAM in the following selected PEGs: (A) PEG_10 in clone 88_1G, (B) PEG_18 in clone 88_1G, (C) PEG_20 in clone 88_1G, (D) PEG_11 in clone 102_5A and (E) PEG_13 in clone 102_5A.

(A)

EMBL-EBI

HOME | SEARCH | BROWSE | FTP | HELP | ABOUT

Pfam

keyword search Go

Sequence search results

Show the detailed description of this results page.

We found **14** Pfam-A matches to your search sequence (there were **no** significant matches)

Show the search options and sequence that you submitted.

Return to the search form to look for Pfam domains on a new sequence.

Insignificant Pfam-A Matches

Show or hide all alignments.

Family	Description	Entry type	Clan	Envelope		Alignment		HMM		HMM length	Bit score	E-value	Predicted active sites	Show/hide alignment
				Start	End	Start	End	From	To					
DUF6290	Family of unknown function (DUF6290)	Family	CL0057	13	88	17	47	**5**	**35**	72	18.8	0.0015	n/a	Show
RHH_1	Ribbon-helix-helix protein, copG family	Domain	CL0057	48	73	48	67	1	**20**	39	4.4	41	n/a	Hide

```
#HMM      rvsisldeellerLdelara
#MATCH    r+ i l ee+++++ el ++
#PP       89999*********9999776
#SEQ      RIGINLTEEEKKTVAELEKR
```

Family	Description	Entry type	Clan	Envelope Start	Envelope End	Alignment Start	Alignment End	HMM From	HMM To	HMM length	Bit score	E-value	Predicted active sites	Show/hide alignment
Ax_dynein_light	Axonemal dynein light chain	Family	n/a	12	89	18	88	**86**	**155**	187	18.6	0.0015	n/a	Show
FapA	Flagellar Assembly Protein A beta soleno ...	Repeat	CL0268	3	89	43	87	**140**	**183**	272	16.8	0.0046	n/a	Show
YPEB	YpeB sporulation	Domain	CL0121	21	88	50	84	**109**	**143**	361	15.7	0.0078	n/a	Show
Prefoldin_2	Prefoldin subunit	Coiled	CL0200	38	89	58	88	**61**	**91**	106	16.0	0.0093	n/a	Show
Cas_Cas02710	CRISPR-associated protein (Cas_Cas02710)	Family	CL0236	3	89	8	88	**151**	**225**	380	13.6	0.024	n/a	Show
DUF1002	Protein of unknown function (DUF1002)	Family	n/a	1	85	4	84	**139**	**219**	220	14.2	0.027	n/a	Show
Phage_GPO	Phage capsid scaffolding protein (GPO) s ...	Family	n/a	1	88	11	88	**169**	**246**	271	13.7	0.038	n/a	Show
PP_kinase_N	Polyphosphate kinase N-terminal domain	Domain	n/a	5	89	8	65	**36**	**96**	107	12.4	0.16	n/a	Show
ZapA	Cell division protein ZapA	Family	n/a	1	89	4	88	**13**	**87**	88	13.0	0.12	n/a	Show
MCU	Mitochondrial calcium uniporter	Family	n/a	3	89	25	88	**37**	**84**	177	12.4	0.14	n/a	Show
Nas2_N	Nas2 N_terminal domain	Domain	n/a	61	89	62	85	**2**	**25**	79	11.0	0.35	n/a	Show
DUF5320	Family of unknown function (DUF5320)	Family	n/a	31	85	55	85	**70**	100	100	11.8	0.47	n/a	Show

(B)

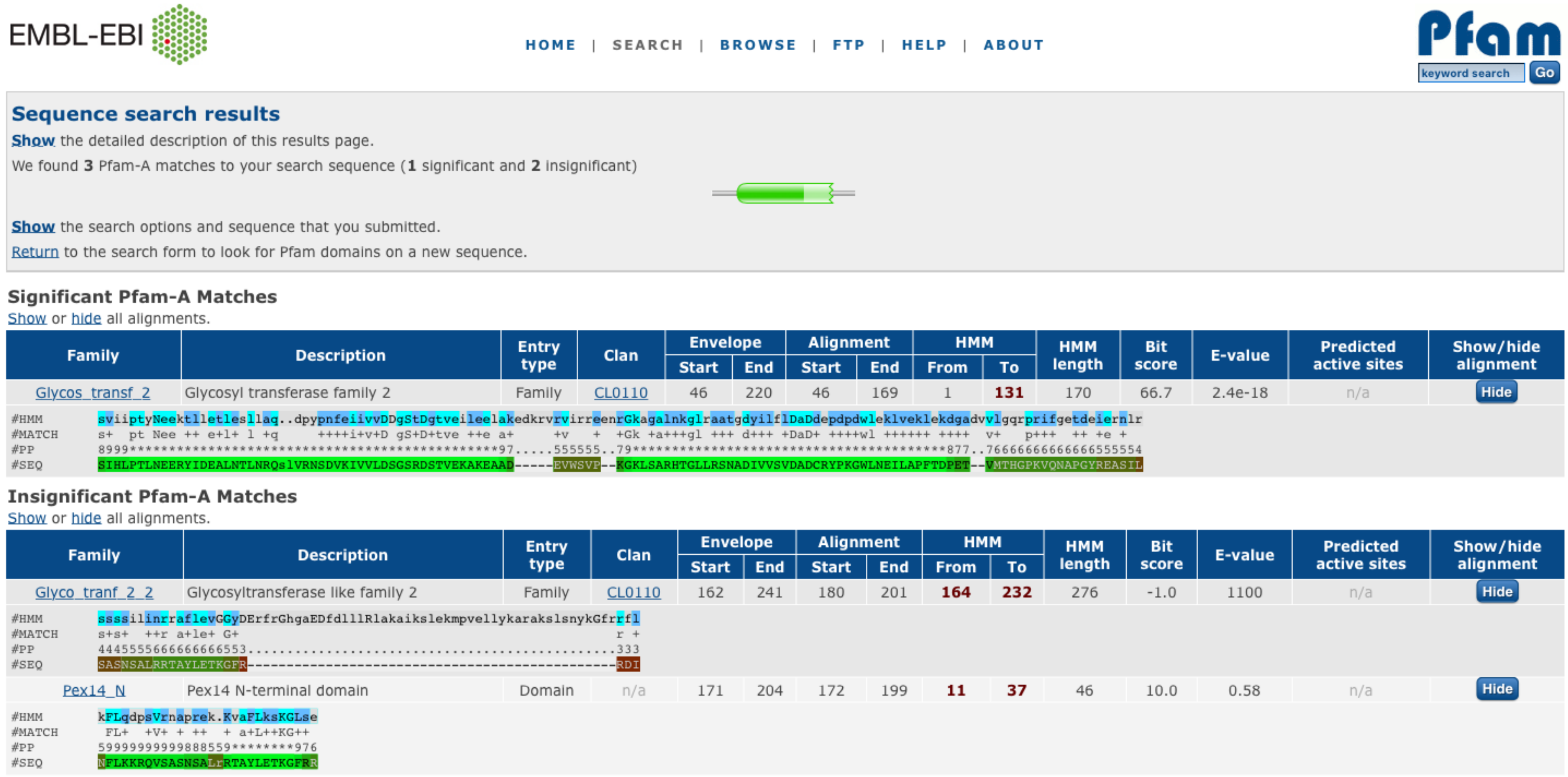

EMBL-EBI

HOME | SEARCH | BROWSE | FTP | HELP | ABOUT

Pfam

keyword search Go

Sequence search results

Show the detailed description of this results page.

We found **3** Pfam-A matches to your search sequence (**1** significant and **2** insignificant)

Show the search options and sequence that you submitted.

Return to the search form to look for Pfam domains on a new sequence.

Significant Pfam-A Matches

Show or hide all alignments.

Family	Description	Entry type	Clan	Envelope		Alignment		HMM		HMM length	Bit score	E-value	Predicted active sites	Show/hide alignment
				Start	End	Start	End	From	To					
Glycos_transf_2	Glycosyl transferase family 2	Family	CL0110	46	220	46	169	1	**131**	170	66.7	2.4e-18	n/a	Hide

```
#HMM    sviiptyNeektlletlesllaq..dpypnfeiivvDDgStDgtveileelakedkrvrvirreenrGkagalnkglraatgdyilflDaDdepdpdwleklveklekdgadvvlgqrprifgetdeiernlr
#MATCH  s+  pt Nee ++ e+l+ l +q      ++++i+v+D gS+D+tve ++e a+      +v    +  +Gk +a+++gl +++ d+++ +DaD+ ++++wl ++++++ ++++  v+    p+++  ++ +e +
#PP     8999***********************************************97.....555555..79****************************************877..76666666666666555554
#SEQ    SIHLPTLNEERYIDEALNTLNRQslVRNSDVKIVVLDSGSRDSTVEKAKEAAD-----EVWSVP--KGKLSARHTGLLRSNADIVVSVDADCRYPKGWLNEILAPFTDPET--VMTHGPKVQNAPGYREASIL
```

Insignificant Pfam-A Matches

Show or hide all alignments.

Family	Description	Entry type	Clan	Envelope		Alignment		HMM		HMM length	Bit score	E-value	Predicted active sites	Show/hide alignment
				Start	End	Start	End	From	To					
Glyco_tranf_2_2	Glycosyltransferase like family 2	Family	CL0110	162	241	180	201	**164**	**232**	276	-1.0	1100	n/a	Hide
Pex14_N	Pex14 N-terminal domain	Domain	n/a	171	204	172	199	**11**	**37**	46	10.0	0.58	n/a	Hide

Glyco_tranf_2_2:

```
#HMM    ssssilinrraflevGGyDErfrGhgaEDfdlllRlakaikslekmpvellykarakslsnykGfrrfl
#MATCH  s+s+  ++r a+le+ G+                                                r +
#PP     4445555666666666553...............................................333
#SEQ    SASNSALRRTAYLETKGFR-----------------------------------------------RDI
```

Pex14_N:

```
#HMM    kFLqdpsVrnaprek.KvaFLksKGLse
#MATCH   FL+   +V+ + ++   + a+L++KG++
#PP     59999999999888559********976
#SEQ    NFLKKRQVSASNSALrRTAYLETKGFRR
```

(C)

EMBL-EBI

HOME | SEARCH | BROWSE | FTP | HELP | ABOUT

Pfam

keyword search Go

Sequence search results

Show the detailed description of this results page.

We found **1** Pfam-A match to your search sequence (**all** significant)

Peptidase_S8

Show the search options and sequence that you submitted.

Return to the search form to look for Pfam domains on a new sequence.

Significant Pfam-A Matches

Show or hide all alignments.

Family	Description	Entry type	Clan	Envelope Start	Envelope End	Alignment Start	Alignment End	HMM From	HMM To	HMM length	Bit score	E-value	Predicted active sites	Show/hide alignment
Peptidase_S8	Subtilase family	Domain	n/a	38	308	55	300	**36**	**276**	284	103.6	1.2e-29	n/a	Hide

```
#HMM   vddvnewneednnnnvlddngHGthvAGiiaangnngkg..vtGvapeakllalkv.dgsgsgstenaikaieraa......nngadvinnSlGedsgggslskelkkaiek.aekkgalfVaaaGNd.....ggnndstvgyPasyknviaVgavd...nednlasfSsygpns..dgalkpdivapGgtitysvnlnsklnedtsanfrqnydsmsGTSmAtPfvsgaaAllksafpkltpee.vkallnntatdlgdpg
#MATCH  ++  ++n +d++ + +d +gHG+ + Gi+a+     ++g   +Gvap+ +l+++kv +++g+g+t+ +i+a++ ++       + +  v+nnS+G +  +g + ++l ka+++ a+  ++ +V+aaGN       +g +d+ ++ P s +nv++V+a+       ++l  +Ss+g +     g+ kpd++ap        ++++    +               + +++   GTS A+P+v+ga+Al+  a +    p++ v ++l  ta    + ++
#PP    5667899999999*************888885.5666689****************************************98888899*******88889999999******999999**********9999888899999***************5554567888999997777789**********8.....22222233.......78899*********************88886655448888888888...4455
#SEQ   GRRMDGYNATDEEGPWSDGHGHGSYTLGIMAGDR-RTQGvdKGGVAPGTDLYPVKVlNSKGEGTTGMVIEALDAVIalaennPGRPVVVNNSWGISRCSGLCGSPLTKAVSRvADVPNVYMVFAAGNMgmkcgQGCSDTGISGPNSLNNVLTVAATGrngYPEKLHRYSSRGGEEasCGSRKPDVAAPI-----YGTVPWGC-------GARDIGNLGGTSGACPHVAGAVALATAAANGVPPASdVFDALRETA---EGKK
```

(D)

EMBL-EBI

HOME | SEARCH | BROWSE | FTP | HELP | ABOUT

Pfam

keyword search Go

Sequence search results

Show the detailed description of this results page.

We found **2** Pfam-A matches to your search sequence (**1** significant and **1** insignificant)

Peptidase_S8

Show the search options and sequence that you submitted.

Return to the search form to look for Pfam domains on a new sequence.

Significant Pfam-A Matches

Show or hide all alignments.

Family	Description	Entry type	Clan	Envelope Start	Envelope End	Alignment Start	Alignment End	HMM From	HMM To	HMM length	Bit score	E-value	Predicted active sites	Show/hide alignment
Peptidase_S8	Subtilase family	Domain	n/a	178	387	198	387	**76**	284	284	71.0	1.0e-19	n/a	Hide

```
#HMM   tGvapeakllalkv.dgsgsgstenaikaieraa....nngadvinnSlGedsgggslskelkkaiek.aekkgalfVaaaGNdggnndstvgyPasyknviaVgavdnednlasfSsygpnsdgalkpdivapGgtitysvnlnsklnedtsanfrqnydsmsGTSmAtPfvsgaaAllksafpkltpeevkalllntatdlgdpgvdraqGvG
#MATCH + +apea+++ +k+ +++g g  +++ika+er+a    n++ d +n+SlG     +  + ++l+ a +  + + ++ +V+aaGN  g+++ +  +Pa+  +++aVga + e+++a+fSs+g ++      d v +G              +s +  ++++++ GTS AtP   +a      +s  +  + ++ +++ l +   d g +g+d a G+G
#PP    678*************************************************...7777888788888888777789**********.**************************************777......******..............3578899**************9888877777777777777777776.66666666666666
#SEQ   SKLAPEAEIITYKAlGAKGLGRISDVIKALERIAnlpeNKKPDLVNLSLG---VPPAIVSPLSIAADAlDSRFNVPVVCAAGNA-GPSAPSAMQPATGSETVAVGALNAEGKVATFSSRGRSV------DTVTYG--------------VVYSYWGGEGIEKVVGTSIATPIFGAAYLDWLSGIKGSNIDDRRMAALSSI-DMGPEGWDPASGYG
```

Insignificant Pfam-A Matches

Show or hide all alignments.

Family	Description	Entry type	Clan	Envelope Start	Envelope End	Alignment Start	Alignment End	HMM From	HMM To	HMM length	Bit score	E-value	Predicted active sites	Show/hide alignment
Peptidase_S8	Subtilase family	Domain	n/a	153	193	153	164	1	**12**	284	-1.9	1700	n/a	Hide

```
#HMM   gkgvvvavlDtG
#MATCH g+gv vav+D+G
#PP    68999*****99
#SEQ   GEGVRVAVVDSG
```

(E)

EMBL-EBI

HOME | SEARCH | BROWSE | FTP | HELP | ABOUT

Pfam

keyword search Go

Sequence search results

Show the detailed description of this results page.

We found **1** Pfam-A match to your search sequence (there were **no** significant matches)

Show the search options and sequence that you submitted.

Return to the search form to look for Pfam domains on a new sequence.

Insignificant Pfam-A Matches

Show or hide all alignments.

Family	Description	Entry type	Clan	Envelope		Alignment		HMM		HMM length	Bit score	E-value	Predicted active sites	Show/hide alignment
				Start	End	Start	End	From	To					
HTH_Tnp_1	Transposase	Family	CL0123	14	46	17	45	**43**	**71**	75	13.6	0.066	n/a	Hide

```
#HMM      Wrkqyregeealpaeseltaleeeirelk
#MATCH    Wr+++ eg+ +  a ++l  +ee++r+L+
#PP       ***************************97
#SEQ      WRRKMVEGMNVAVAHDRLERIEERLRRLE
```

Table S1: Annotation of all PEGs after filtering and trimming of the putative orphan biosynthetic gene clusters on 88-1G clone insert.

Contig	Start	Stop	PEG	RAST annotation	Best hit from psi-BLAST	Query coverage	E-value	Identity%	Accession number	PEG length (bp)	MIBIG hit	BGC reference in MIBiG	Organism	Score	E-Value	Other
contig_11	51	194	1	hypothetical protein	LysR family transcriptional regulator [Hydrocarboniphaga effusa]	93%	4.9	36%	gi|494335651|WP_007184865.1	144	BGC0001024|c1|1388-13399|+|no_locus_tag|MtaB|AAF19810.1 Length=4003	Myxothiazol biosynthetic gene cluster	*Stigmatella aurantiaca* DW4/3-1 Bacteria; Proteobacteria; Deltaproteobacteria; Myxococcales; Cystobacterineae; Cystobacteraceae; *Stigmatella*.	25.8	1.3	Identities = 10/39 (26%), Positives = 19/39 (49%), Gaps = 0/39 (0%) Frame = -2
contig_11	199	909	2	hypothetical protein	cell surface glycoprotein [Natronomonas pharaonis]	39%	0.087	31%	gi|499643284|WP_011324018.1	711	BGC0000192|c1|4082-4867|-|no_locus_tag|AknA|AAF70104.1 Length=261	Aclacinomycin biosynthetic gene cluster	*Streptomyces galilaeus* Bacteria; Actinobacteria; Actinobacteridae; Actinomycetales; Streptomycineae; Streptomycetaceae; *Streptomyces*.	28.5	4.9	Identities = 20/65 (31%), Positives = 30/65 (46%), Gaps = 0/65 (0%) Frame = +1
contig_11	997	2142	3	hypothetical protein	hypothetical protein [Natronobacterium texcoconense]	88%	2e-07	24%	gi|1222330301|WP_090383325.1	1146	BGC0001396|c1|62377-64848|+|no_locus_tag|AlmRII|ANC94980.1 Length=823	Aldgamycin biosynthetic gene cluster	*Streptomyces* sp. A1(2016) Bacteria; Actinobacteria; Streptomycetales; Streptomycetaceae; *Streptomyces*.	32.3	0.72	Identities = 21/71 (30%), Positives = 34/71 (48%), Gaps = 6/71 (8%) Frame = +1
contig_11	2547	2158	4	hypothetical protein	sulfite exporter TauE/SafE family protein [Nocardiopsis halotolerans]	48%	1.2	39%	gi|516142320|WP_017572900.1	390	BGC0000109|c1|1013-3781|-|no_locus_tag|transcriptional_regulatory_protein|BAF85834.1 Length=922	Nemadectin biosynthetic gene cluster	*Streptomyces cyaneogriseus* subsp. noncyanogenus Bacteria; Actinobacteria; Actinobacteridae; Actinomycetales; Streptomycineae; Streptomycetaceae; *Streptomyces*.	29.6	0.64	Identities = 27/99 (27%), Positives = 39/99 (39%), Gaps = 5/99 (5%) Frame = +1
contig_127	259	408	5	hypothetical protein	Uncharacterised protein [uncultured Blautia sp.]	93%	0.007	52%	gi|1052846480|SCI34226.1	150	BGC0001284|c1|1-5292|+|no_locus_tag|polyketide_synthase|AKN45693.1 Length=1763	Alternariol biosynthetic gene cluster	*Parastagonospora nodorum* SN15 Eukaryota; Fungi; Dikarya; Ascomycota; Pezizomycotina; Dothideomycetes; Pleosporomycetidae; Pleosporales; Pleosporineae; Phaeosphaeriaceae; *Parastagonospora*.	25.8	1.3	Identities = 7/17 (41%), Positives = 12/17 (71%), Gaps = 0/17 (0%) Frame = -1

<table>
<tr><th>Contig</th><th>Start</th><th>Stop</th><th>PEG</th><th>RAST annotation</th><th>Best hit from psi-BLAST</th><th>Query coverage</th><th>E-value</th><th>Identity%</th><th>Accession number</th><th>PEG length (bp)</th><th>MIBIG hit</th><th>BGC reference in MIBiG</th><th>Organism</th><th>Score</th><th>E-Value</th><th>Other</th></tr>
<tr><td>contig_13</td><td>205</td><td>753</td><td>6</td><td>hypothetical protein</td><td>serine/threonine protein phosphatase [Halopelagius longus]</td><td>86%</td><td>7e-12</td><td>29%</td><td>gi|1224515647|WP_092536451.1</td><td>549</td><td>BGC0000268|c1|72198-73847|-|no_locus_tag|putative_secreted_protein|CAH10133.1
Length=549</td><td>Sch47554 / Sch47555 biosynthetic gene cluster</td><td>Streptomyces sp. SCC 2136
Bacteria; Actinobacteria; Actinobacteridae; Actinomycetales; Streptomycineae; Streptomycetaceae; Streptomyces.</td><td>30.4</td><td>0.84</td><td>Identities = 16/42 (38%), Positives = 23/42 (55%), Gaps = 3/42 (7%)
Frame = +1</td></tr>
<tr><td>contig_13</td><td>759</td><td>2198</td><td>7</td><td>MJ0042 family finger-like protein</td><td>hypothetical protein MTR_2g090985 [Medicago truncatula]</td><td>99%</td><td>7e-16</td><td>25%</td><td>gi|922393585|XP_013465200.1</td><td>1440</td><td>BGC0001379|c1|20163-21161|-|no_locus_tag|oxidoreductase|BAU50940.1
Length=332</td><td>BD-12 biosynthetic gene cluster</td><td>Streptomyces luteocolor
Bacteria; Actinobacteria; Streptomycetales; Streptomycetaceae; Streptomyces.</td><td>31.2</td><td>1.6</td><td>Identities = 14/26 (54%), Positives = 19/26 (73%), Gaps = 1/26 (4%)
Frame = +1</td></tr>
<tr><td>contig_13</td><td>2257</td><td>2427</td><td>8</td><td>hypothetical protein</td><td>hypothetical protein [Clostridia bacterium UC5.1-1C12]</td><td>58%</td><td>9.8</td><td>42%</td><td>gi|983544544|WP_060696140.1</td><td>171</td><td>BGC0000422|c1|41474-42574|+|no_locus_tag|SAM-dependent_methyltransferase|ABI22137.1
Length=366</td><td>Saframycin A biosynthetic gene cluster</td><td>Streptomyces lavendulae
Bacteria; Actinobacteria; Actinobacteridae; Actinomycetales; Streptomycineae; Streptomycetaceae; Streptomyces.</td><td>25.8</td><td>1.7</td><td>Identities = 7/24 (29%), Positives = 15/24 (63%), Gaps = 0/24 (0%)
Frame = +1</td></tr>
<tr><td>contig_13</td><td>2636</td><td>2430</td><td>9</td><td>hypothetical protein</td><td>hypothetical protein Egran_04870 [Elaphomyces granulatus]</td><td>80%</td><td>1.8</td><td>33%</td><td>gi|1227549077|OXV07365.1</td><td>207</td><td>BGC0001069|c1|5072-22603|+|no_locus_tag|polyketide_synthase|AAY89049.1
Length=5843</td><td>Piericidin A1 biosynthetic gene cluster</td><td>Streptomyces sp. SCSIO 03032
Bacteria; Actinobacteria; Actinobacteridae; Actinomycetales; Streptomycineae; Streptomycetaceae; Streptomyces.</td><td>26.2</td><td>2.3</td><td>Identities = 14/54 (26%), Positives = 28/54 (52%), Gaps = 0/54 (0%)
Frame = -2</td></tr>
<tr><td>contig_13</td><td>2702</td><td>2971</td><td>10</td><td>hypothetical protein</td><td>CopG family transcriptional regulator [Natronococcus occultus]</td><td>80%</td><td>0.001</td><td>35%</td><td>gi|505136118|WP_015323220.1</td><td>270</td><td>BGC0000978|c1|444-6074|+|no_locus_tag|polyketide_synthase|ABX60152.1
Length=1876</td><td>Cylindrospermopsin biosynthetic gene cluster</td><td>Cylindrospermopsis raciborskii AWT205
Bacteria; Cyanobacteria; Nostocales; Nostocaceae; Cylindrospermopsis.</td><td>26.2</td><td>4.9</td><td>Identities = 11/37 (30%), Positives = 21/37 (57%), Gaps = 1/37 (3%)
Frame = +2</td></tr>
<tr><td>contig_13</td><td>3288</td><td>2986</td><td>11</td><td>hypothetical protein</td><td>outer membrane lipoprotein carrier protein LolA [Pseudoxanthomonas suwonensis]</td><td>31%</td><td>1.3</td><td>55%</td><td>gi|640336110|WP_024867837.1</td><td>303</td><td>BGC0000154|c1|24057-25227|-|TSTA_117800|1-aminocyclopropane-1-carboxylate_oxidase,_putative|EED18007.1
Length=325</td><td>Stipitatic acid biosynthetic gene cluster</td><td>Talaromyces stipitatus ATCC 10500
Eukaryota; Fungi; Dikarya; Ascomycota; Pezizomycotina; Eurotiomycetes; Eurotiomycetidae; Eurotiales; Trichocomaceae; Talaromyces.</td><td>29.3</td><td>0.5</td><td>Identities = 21/62 (34%), Positives = 32/62 (52%), Gaps = 6/62 (10%)
Frame = +1</td></tr>
<tr><td>contig_13</td><td>3882</td><td>3379</td><td>12</td><td>hypothetical protein</td><td>hypothetical protein PFHG_02497 [Plasmodium falciparum HB3]</td><td>86%</td><td>4e-05</td><td>27%</td><td>gi|914547632|KOB60765.1</td><td>504</td><td>BGC0000464|c1|77134-83853|+|XDD1_2287|non-ribosomal_peptide_synthetase|CDG17986.1
Length=2239</td><td>Xenoamicins biosynthetic gene cluster</td><td>Xenorhabdus doucetiae
Bacteria; Proteobacteria; Gammaproteobacteria; Enterobacteriales; Enterobacteriaceae; Xenorhabdus.</td><td>28.9</td><td>2.5</td><td>Identities = 18/56 (32%), Positives = 30/56 (54%), Gaps = 3/56 (5%)
Frame = +1</td></tr>
</table>

<table>
<tr><th>Contig</th><th>Start</th><th>Stop</th><th>PEG</th><th>RAST annotation</th><th>Best hit from psi-BLAST</th><th>Query coverage</th><th>E-value</th><th>Identity%</th><th>Accession number</th><th>PEG length (bp)</th><th>MIBIG hit</th><th>BGC reference in MIBiG</th><th>Organism</th><th>Score</th><th>E-Value</th><th>Other</th></tr>
<tr><td>contig_13</td><td>4011</td><td>3895</td><td>13</td><td>hypothetical protein</td><td>sentrin/SUMO-specific protease [Diplocarpon rosae]</td><td>68%</td><td>4.5</td><td>58%</td><td>gi|1244198543|PBP23272.1</td><td>117</td><td>BGC0001096|c1|1164-2939|+|no_locus_tag|hypothetical_protein|ADH01477.1
Length=591</td><td>FR901464 biosynthetic gene cluster</td><td>Pseudomonas sp. 2663
Bacteria; Proteobacteria; Gammaproteobacteria; Pseudomonadales; Pseudomonadaceae; Pseudomonas.</td><td>25.8</td><td>0.76</td><td>Identities = 11/17 (65%), Positives = 13/17 (76%), Gaps = 0/17 (0%)
Frame = +1</td></tr>
<tr><td>contig_13</td><td>4642</td><td>4073</td><td>14</td><td>hypothetical protein</td><td>flagellar protein D [Thermococcus celer]</td><td>48%</td><td>0.036</td><td>34%</td><td>gi|1214752275|WP_088863581.1</td><td>570</td><td>BGC0000133|c1|77436-79640|-|no_locus_tag|exodeoxyribonuclease|AFI57030.1
Length=734</td><td>Quartromicin biosynthetic gene cluster</td><td>Amycolatopsis orientalis
Bacteria; Actinobacteria; Actinobacteridae; Actinomycetales; Pseudonocardineae; Pseudonocardiaceae; Amycolatopsis.</td><td>32.3</td><td>0.21</td><td>Identities = 20/51 (39%), Positives = 29/51 (57%), Gaps = 4/51 (8%)
Frame = +1</td></tr>
<tr><td>contig_13</td><td>4930</td><td>4715</td><td>15</td><td>hypothetical protein</td><td>No significant similarity found.</td><td></td><td></td><td></td><td></td><td>216</td><td>BGC0000142|c1|14735-36367|-|Strop_2768|beta-ketoacyl_synthase|ABP55210.1
Length=7210</td><td>Salinilactam biosynthetic gene cluster</td><td>Salinispora tropica CNB-440
Bacteria; Actinobacteria; Actinobacteridae; Actinomycetales; Micromonosporineae; Micromonosporaceae; Salinispora.</td><td>26.6</td><td>1.7</td><td>Identities = 10/28 (36%), Positives = 16/28 (57%), Gaps = 0/28 (0%)
Frame = +2</td></tr>
<tr><td>contig_34</td><td>257</td><td>129</td><td>16</td><td>hypothetical protein</td><td>hypothetical protein M918_24845 [Clostridium sp. BL8</td><td>95%</td><td>6e-13</td><td>80%</td><td>gi|530690040|EQB88155.1</td><td>129</td><td>BGC0000393|c1|2462-45286|+|MXAN_3779|non-ribosomal_peptide_synthetase/polyketide_synthase|ABF87031.1
Length=14274</td><td>Myxoprincomide biosynthetic gene cluster</td><td>Myxococcus xanthus DK 1622
Bacteria; Proteobacteria; Deltaproteobacteria; Myxococcales; Cystobacterineae; Myxococcaceae; Myxococcus.</td><td>23.5</td><td>7.7</td><td>Identities = 9/15 (60%), Positives = 10/15 (67%), Gaps = 0/15 (0%)
Frame = -1</td></tr>
<tr><td>contig_753</td><td>476</td><td>60</td><td>17</td><td>hypothetical protein</td><td>phosphopentomutase [Paenibacillus odorifer]</td><td>50%</td><td>0.003</td><td>36%</td><td>gi|1133910987|WP_076284511.1</td><td>417</td><td>BGC0001232|c1|18438-33497|-|BI06_RS39070|polyketide_synthase|WP_043476519.1
Length=5019</td><td>Leucanicidin biosynthetic gene cluster</td><td>Kitasatospora sp. MBT66
Bacteria; Actinobacteria; Streptomycetales; Streptomycetaceae; Kitasatospora.</td><td>31.2</td><td>0.25</td><td>Identities = 27/107 (25%), Positives = 48/107 (45%), Gaps = 4/107 (4%)
Frame = +1</td></tr>
<tr><td>contig_8</td><td>1248</td><td>466</td><td>18</td><td>glycosyltransferase</td><td>hypothetical protein AUJ13_03040 [Candidatus Micrarchaeota archaeon CG1_02_49_24]</td><td>81%</td><td>6e-21</td><td>30%</td><td>gi|1101062238|OIO23950.1</td><td>783</td><td>BGC0000644|c1|5545-6276|-|no_locus_tag|glucosyl_transferase|ABD24403.1
Length=243</td><td>Carotenoid biosynthetic gene cluster</td><td>Dietzia sp. CQ4
Bacteria; Actinobacteria; Actinobacteridae; Actinomycetales; Corynebacterineae; Dietziaceae; Dietzia.</td><td>62.4</td><td>2.00E-11</td><td>Identities = 59/191 (31%), Positives = 93/191 (49%), Gaps = 20/191 (10%)
Frame = +1</td></tr>
<tr><td>contig_8</td><td>1291</td><td>1500</td><td>19</td><td>hypothetical protein</td><td>serine protease [Geodermatophilus sp. Leaf369]</td><td>95%</td><td>0.079</td><td>36%</td><td>gi|946835434|WP_055758537.1</td><td>210</td><td>BGC0001008|c1|64077-69911|-|no_locus_tag|enediyne_polyketide_synthase|AAQ17110.2
Length=1944</td><td>Maduropeptin biosynthetic gene cluster</td><td>Actinomadura madurae
Bacteria; Actinobacteria; Actinobacteridae; Actinomycetales; Streptosporangineae; Thermomono-sporaceae; Actinomadura.</td><td>29.3</td><td>0.17</td><td>Identities = 15/39 (38%), Positives = 19/39 (49%), Gaps = 0/39 (0%)
Frame = +1</td></tr>
</table>

Contig	Start	Stop	PEG	RAST annotation	Best hit from psi-BLAST	Query coverage	E-value	Identity%	Accession number	PEG length (bp)	MIBIG hit	BGC reference in MIBiG	Organism	Score	E-Value	Other
contig_8	1548	2609	20	peptidase S8 and S53	serine protease AprX [Bradyrhizobium erythrophlei]	79%	6e-26	34%	gi|1119071599|SIO25423.1	1062	BGC0000596|c1|8821-12483|+|SSPG_05087|peptidase_S8|WP_003976359.1 Length=1220	SLI-2138 biosynthetic gene cluster	*Streptomyces lividans* TK24 Bacteria; Actinobacteria; Actinobacteridae; Actinomycetales; Streptomycineae; Streptomycetaceae; *Streptomyces*.	47	2.00E-05	Identities = 54/201 (27%), Positives = 86/201 (43%), Gaps = 21/201 (10%) Frame = +1
contig_8	2907	2677	21	hypothetical protein	PREDICTED: collectrin [Anolis carolinensis]	59%	0.18	42%	gi|327268311|XP_003218941.1	231	BGC0000030|c1|13737-16100|-|FFUJ_06746|bikaverin_cluster-transcription_factor|CCT67995.1 Length=743	Bikaverin biosynthetic gene cluster	*Fusarium fujikuroi* IMI 58289 Eukaryota; Fungi; Dikarya; Ascomycota; Pezizomycotina; Sordariomycetes; Hypocreomycetidae; Hypocreales; Nectriaceae; *Fusarium*; *Fusarium fujikuroi* species complex.	26.9	1.4	Identities = 15/52 (29%), Positives = 25/52 (48%), Gaps = 9/52 (17%) Frame = -1
contig_8	2947	3159	22	hypothetical protein	thiol reductant ABC exporter subunit CydD [Paenibacillus apiarius]	88%	0.29	30%	gi|1199704204|WP_087434714.1	213	BGC0001019|c1|16327-20982|+|no_locus_tag|polyketide_synthase|AHB82053.1 Length=1551	Microsclerodermins biosynthetic gene cluster	*Sorangium cellulosum* Bacteria; Proteobacteria; Deltaproteobacteria; Myxococcales; Sorangiineae; Polyangiaceae; *Sorangium*.	25	5.7	Identities = 15/34 (44%), Positives = 16/34 (47%), Gaps = 0/34 (0%) Frame = +1

Three annotation tools were used: RAST (Aziz et al., 2008), psi-BLAST (Altschul et al., 1997) and BLASTX against MIBiG curated sequences (Medema et al., 2015) are depicted.

Table S2: Annotation of all PEGs after filtering and trimming of the putative orphan biosynthetic gene clusters on 102-5A clone insert.

Contig	Start	Stop	PEG	RAST annotation	Best hit from psi-BLAST	Query covera	E-value	Identity	Accession number	PEG length (bp)	MIBIG hit	BGC reference in MIBiG	Organism	Score	E-Value	Other
contig_1	79	447	1	hypothetical protein	No significant similarity found.					369	BGC0000151|c1|58260-77261|-|SAMR0474|putative_modular_polyketide_synthase|CAJ88184.1 Length=6333	Stambomycin biosynthetic gene cluster	*Streptomyces ambofaciens* ATCC 23877 Bacteria; Actinobacteria; Actinobacteridae; Actinomycetales; Streptomycineae; Streptomycetaceae; *Streptomyces*.	27.3	4.4	Identities = 22/62 (35%), Positives = 29/62 (47%), Gaps = 4/62 (6%) Frame = +1
contig_1	449	658	2	hypothetical protein	4Fe-4S dicluster domain-containing protein [Bacillus alveayuensis]	72%	0.059	35%	gi|765542597|WP_044749318.1	210	BGC0001380|c1|11917-12837|+|no_locus_tag|UTP-glucose-1-phosphate_uridylyltransferase|ALI93250.1 Length=306	Salecan biosynthetic gene cluster	*Agrobacterium* sp. ZX09 Bacteria; Proteobacteria; Alphaproteobacteria; Rhizobiales; Rhizobiaceae; Rhizobium/Agrobacterium group; *Agrobacterium*.	28.5	0.3	Identities = 12/33 (36%), Positives = 22/33 (67%), Gaps = 0/33 (0%) Frame = -3
contig_1	662	1534	3	Cell division protein FtsW	hypothetical protein B6U84_05395 [Candidatus Bathyarchaeota archaeon ex4484_40]	64%	1e-07	28%	gi|1231981811|OYT43840.1	873	BGC0001413|c1|7-3810|-|no_locus_tag|CysT|AKP45383.1 Length=1267	Cystobactamide biosynthetic gene cluster	*Cystobacter* sp. Cbv34 Bacteria; Proteobacteria; Deltaproteobacteria; Myxococcales; Cystobacterineae; Cystobacteraceae; *Cystobacter*.	35	0.067	Identities = 36/117 (31%), Positives = 54/117 (46%), Gaps = 21/117 (18%) Frame = +1
contig_1	1557	2489	4	hypothetical protein	Uncharacterized protein LW93_12271 [Fusarium fujikuroi]	41%	4e-06	33%	gi|829112408|KLO88856.1	933	BGC0000369|c1|27532-38469|+|no_locus_tag|non-ribosomal_peptide_synthase|AHZ20774.1 Length=3645	Hassallidins biosynthetic gene cluster	*Anabaena* sp. Syke748 Bacteria; Cyanobacteria; Nostocales; Nostocaceae; *Anabaena*.	33.9	0.18	Identities = 16/46 (35%), Positives = 25/46 (54%), Gaps = 0/46 (0%) Frame = -1
contig_1	2599	4533	5	hypothetical protein	hypothetical protein US59_C0012G0019 [Candidatus Levybacteria bacterium GW2011_GWB1_37_8]	30%	9e-14	30%	gi|818361484|KKQ42274.1	1935	BGC0000326|c1|47536-48684|+|no_locus_tag|prephenate_dehydrogenase|AAK81837.1 Length=382	Complestatin biosynthetic gene cluster	*Streptomyces lavendulae* Bacteria; Actinobacteria; Actinobacteridae; Actinomycetales; Streptomycineae; Streptomycetaceae; *Streptomyces*.	30	6.8	Identities = 23/79 (29%), Positives = 37/79 (47%), Gaps = 11/79 (14%) Frame = +1

Contig	Start	Stop	PEG	RAST annotation	Best hit from psi-BLAST	Query covera	E-value	Identity	Accession number	PEG length (bp)	MIBIG hit	BGC reference in MIBiG	Organism	Score	E-Value	Other
contig_1	4542	4733	6	hypothetical protein	S9 family peptidase [Nocardia pseudobrasiliensis]	68%	0.79	49%	gi|1056574138|WP_068006013.1	192	BGC0000435|c1|10568-28357|+|PSPTO_2830|non-ribosomal_peptide_synthetase_SyfB|AAO56329.1 Length=5929	Syringafactin biosynthetic gene cluster	*Pseudomonas syringae* pv. tomato str. DC3000 Bacteria; Proteobacteria; Gammaproteobacteria; Pseudomonadales; Pseudomonadaceae; *Pseudomonas*.	28.9	0.21	Identities = 16/37 (43%), Positives = 25/37 (68%), Gaps = 5/37 (14%) Frame = -2
contig_1	4746	4931	7	hypothetical protein	hypothetical protein AKJ51_00470 [candidate divison MSBL1 archaeon SCGC-AAA382A20]	98%	1e-08	40%	gi|985677532|KXB07656.1	186	BGC0001333|c1|14439-16040|+|no_locus_tag|RebC-like_monooxygenase|AEF32100.1 Length=533	BE-54017 biosynthetic gene cluster	uncultured bacterium AB1650 Bacteria; environmental samples.	26.6	1.3	Identities = 9/24 (38%), Positives = 14/24 (58%), Gaps = 0/24 (0%) Frame = -1
contig_1	4928	5950	8	hypothetical protein	PREDICTED: insulin receptor substrate 4 [Myotis lucifugus]	26%	0.15	34%	gi|940712809|XP_014315965.1	1023	BGC0000349|c1|3828-20117|+|SACE_3035|putative_non-ribosomal_peptide_synthetase|CAM02313.1 Length=5429	Erythrochelin biosynthetic gene cluster	*Saccharopolyspora erythraea* NRRL 2338 Bacteria; Actinobacteria; Actinobacteridae; Actinomycetales; Pseudonocardineae; Pseudonocardiaceae; *Saccharopolyspora*.	29.3	5.7	Identities = 32/81 (40%), Positives = 37/81 (46%), Gaps = 7/81 (9%) Frame = -1
contig_1	5952	6236	9	hypothetical protein	hypothetical protein [delta proteobacterium PSCGC 5342]	36%	0.51	47%	gi|654514092|WP_027983256.1	285	BGC0001096|c1|53637-62690|+|no_locus_tag|polyketide_synthase|ADH01488.1 Length=3017	FR901464 biosynthetic gene cluster	*Pseudomonas* sp. 2663 Bacteria; Proteobacteria; Gammaproteobacteria; Pseudomonadales; Pseudomonadaceae; *Pseudomonas*.	28.9	0.53	Identities = 12/39 (31%), Positives = 20/39 (51%), Gaps = 0/39 (0%) Frame = +1
contig_1	6233	6520	10	hypothetical protein	exopolysaccharide biosynthesis protein ExoD [Rhizobium sp. CF097]	77%	0.047	36%	gi|739259268|WP_037122249.1	288	BGC0001096|c1|62806-70794|+|no_locus_tag|type_I_polyketide_synthase|ADH01489.1 Length=2662	FR901464 biosynthetic gene cluster	*Pseudomonas* sp. 2663 Bacteria; Proteobacteria; Gammaproteobacteria; Pseudomonadales; Pseudomonadaceae; *Pseudomonas*.	26.6	3.3	Identities = 14/55 (25%), Positives = 23/55 (42%), Gaps = 10/55 (18%) Frame = +3
contig_1	6517	7809	11	Subtilisin precursor	peptidase S8 [Streptomyces sp. CB02613]	75%	3e-21	28%	gi|1288530123|WP_100563527.1	1293	BGC0000596|c1|8821-12483|+|SSPG_05087|peptidase_S8|WP_003976359.1 Length=1220	SLI-2138 biosynthetic gene cluster	*Streptomyces lividans* TK24 Bacteria; Actinobacteria; Actinobacteridae; Actinomycetales; Streptomycineae; Streptomycetaceae; *Streptomyces*.	52	#######	Identities = 57/212 (27%), Positives = 95/212 (45%), Gaps = 30/212 (14%) Frame = +1

Contig	Start	Stop	PEG	RAST annotation	Best hit from psi-BLAST	Query covera	E-value	Identity	Accession number	PEG length (bp)	MIBIG hit	BGC reference in MIBiG	Organism	Score	E-Value	Other
contig_1	7862	8449	12	hypothetical protein	CDP-diacylglycerol--glycerol-3-phosphate 3-phosphatidyltransferase [Crinalium epipsammum]	90%	0.18	28%	gi|505014458|WP_015201560.1	588	BGC0001323|c1|62414-69860|+|no_locus_tag|terpene_synthase_18|AGK82823.1 Length=770	Monoterpenes-diterpenes biosynthetic gene cluster	Solanum pennellii Eukaryota; Viridiplantae; Streptophyta; Embryophyta; Tracheophyta; Spermatophyta; Magnoliophyta; eudicotyledons; Gunneridae; Pentapetalae; asterids; lamiids; Solanales; Solanaceae; Solanoideae; Solaneae; *Solanum; Lycopersicon*.	28.9	2.9	Identities = 13/46 (28%), Positives = 22/46 (48%), Gaps = 1/46 (2%) Frame = +1
contig_1	8840	8697	13	hypothetical protein	cobalt-precorrin 5A hydrolase [Hyphomicrobium facile]	93%	0.56	46%	gi|1097765764|SFV26573.1	144	BGC0000439|c1|3956-4804|-|no_locus_tag|ABC-type_multidrug_transport_system|AHH53500.1 Length=282	Taromycin biosynthetic gene cluster	*Saccharomonospora* sp. CNQ490 Bacteria; Actinobacteria; Actinobacteridae; Actinomycetales; Pseudonocardineae; Pseudonocardiaceae; *Saccharomonospora*.	24.6	3	Identities = 9/19 (47%), Positives = 10/19 (53%), Gaps = 0/19 (0%) Frame = -1
contig_1	9517	9128	14	hypothetical protein	DUF4142 domain-containing protein [Pedobacter steynii]	63%	0.71	29%	gi|1124311659|WP_074604802.1	390	BGC0001111|c1|43574-49810|+|no_locus_tag|trans-AT_type_I_polyketide_synthase|CCA89327.1 Length=2078	Rhizopodins biosynthetic gene cluster	*Stigmatella aurantiaca* Sg a15 Bacteria; Proteobacteria; Deltaproteobacteria; Myxococcales; Cystobacterineae; Cystobacteraceae; *Stigmatella*.	27.7	3.6	Identities = 14/41 (34%), Positives = 21/41 (51%), Gaps = 3/41 (7%) Frame = +1
contig_1	9962	9519	15	hypothetical protein	hypothetical protein B6U95_00355 [Thermofilum sp. ex4484_82]	54%	1e-05	36%	gi|1231967246|OYT30423.1	444	BGC0001011|c1|43455-64763|+|no_locus_tag|polyketide_synthase|ABC87510.1 Length=7102	Meridamycin biosynthetic gene cluster	*Streptomyces* sp. NRRL 30748 Bacteria; Actinobacteria; Actinobacteridae; Actinomycetales; Streptomycineae; Streptomycetaceae; *Streptomyces*.	29.3	1.5	Identities = 20/83 (24%), Positives = 27/83 (33%), Gaps = 9/83 (11%) Frame = +2
contig_1	11484	10117	16	Phage protein	hypothetical protein OSG_eHP23_00130 [Environmental Halophage eHP-23]	54%	1e-51	37%	gi|383397572|AFH22355.1	1368	BGC0001169|c1|18700-23964|+|no_locus_tag|PieA3|AHE80993.1 Length=1754	Piericidin A1 biosynthetic gene cluster	*Streptomyces* sp. SCSIO 03032 Bacteria; Actinobacteria; Actinobacteridae; Actinomycetales; Streptomycineae; Streptomycetaceae; *Streptomyces*.	32.3	0.94	Identities = 23/80 (29%), Positives = 36/80 (45%), Gaps = 3/80 (4%) Frame = +1

Contig	Start	Stop	PEG	RAST annotation	Best hit from psi-BLAST	Query covera	E-value	Identity	Accession number	PEG length (bp)	MIBIG hit	BGC reference in MIBiG	Organism	Score	E-Value	Other
contig_1	12005	11520	17	Putative preQ0 transporter	MULTISPECIES: hypothetical protein [Desulfitobacterium]	83%	3e-31	47%	gi|517010576|WP_018211389.1	486	BGC0001177|c1|18644-19891|-|no_locus_tag|putative_bifunctional_cyclase/dehydratase|AHL46690.1 Length=415	Mensacaricin biosynthetic gene cluster	*Streptomyces bottropensis* Bacteria; Actinobacteria; Actinobacteridae; Actinomycetales; Streptomycineae; Streptomycetaceae; *Streptomyces*.	30	0.76	Identities = 13/39 (33%), Positives = 23/39 (59%), Gaps = 0/39 (0%) Frame = -2
contig_7	1117	809	18	hypothetical protein	hypothetical protein [Thermoanaerobacterium sp. PSU-2]	60%	7e-10	50%	gi|1184544938|WP_085113370.1	309	BGC0000012|c1|11261-19092|+|no_locus_tag|PKSN_polyketide_synthase_for_alternapyrone_biosynthesis|BAD83684.1 Length=2551	Alternapyrone biosynthetic gene cluster	*Alternaria solani* Eukaryota; Fungi; Dikarya; Ascomycota; Pezizomycotina; Dothideomycetes; Pleosporomycetidae; Pleosporales; Pleosporineae; Pleosporaceae; *Alternaria*.	26.2	6.2	Identities = 9/20 (45%), Positives = 14/20 (70%), Gaps = 0/20 (0%) Frame = +3
contig_7	1791	1135	19	hypothetical protein	hypothetical protein CONLIGDRAFT_635943 [Coniochaeta ligniaria NRRL 30616]	13%	0.40	53%	gi|1102742213|OIW24806.1	657	BGC0000052|c1|25902-36188|+|no_locus_tag|polyketide_synthase_type_I|AAX98185.1 Length=3428	ECO-02301 biosynthetic gene cluster	*Streptomyces aizunensis* Bacteria; Actinobacteria; Actinobacteridae; Actinomycetales; Streptomycineae; Streptomycetaceae; Streptomyces.	29.6	2.4	Identities = 17/48 (35%), Positives = 24/48 (50%), Gaps = 3/48 (6%) Frame = +1
contig_7	2986	1817	20	hypothetical protein	hypothetical protein J07AB56_08720 [Candidatus Nanosalinarum sp. J07AB56]	98%	8e-14	31%	gi|339756561|EGQ40144.1	1170	BGC0001099|c1|6732-30773|+|no_locus_tag|Bat2|ADD82940.1 Length=8013	Kalimantacin / batumin biosynthetic gene cluster	*Pseudomonas fluorescens* Bacteria; Proteobacteria; Gammaproteobacteria; Pseudomonadales; Pseudomonadaceae; *Pseudomonas*.	30	4.1	Identities = 14/55 (25%), Positives = 29/55 (53%), Gaps = 2/55 (4%) Frame = +1
contig_7	3679	3071	21	hypothetical protein	hypothetical protein pSs1_00252 [Shigella phage pSs-1]	72%	0.57	29%	gi|730984967|YP_009111060.1	609	BGC0001107|c1|2321-8444|+|no_locus_tag|PtzC|AFK83819.1 Length=2041	Patellazole biosynthetic gene cluster	*Candidatus Endolissoclinum faulkneri* L5 Bacteria; Proteobacteria; Alphaproteobacteria; Rhodospirillales; Rhodospirillaceae; Candidatus *Endolissoclinum*.	28.1	6.5	Identities = 15/38 (39%), Positives = 22/38 (58%), Gaps = 3/38 (8%) Frame = -1

Three annotation tools were used: RAST (Aziz et al., 2008), psi-BLAST (Altschul et al., 1997) and BLASTX against MIBiG curated sequences (Medema et al., 2015) are depicted.
